# Within-subject variation of HbA1c: A systematic review and meta-analysis

**DOI:** 10.1371/journal.pone.0289085

**Published:** 2023-08-02

**Authors:** Alex Gough, Alice Sitch, Erica Ferris, Tom Marshall

**Affiliations:** Institute of Applied Health Research, College of Medical and Dental Sciences, University of Birmingham, Birmingham, United Kingdom; University of Campania Luigi Vanvitelli: Universita degli Studi della Campania Luigi Vanvitelli, ITALY

## Abstract

**Background:**

Glycosylated haemoglobin (HbA1c) measurement is used to diagnose and to guide treatment of diabetes mellitus. Within-subject variability in measured HbA1c affects its clinical utility and interpretation, but no comprehensive systematic review has described within-subject variability.

**Methods:**

A systematic review and meta-analysis was performed of within-subject variability of HbA1c. Multiple databases were searched from inception to November 2022 for follow-up studies of any design in adults or children, with repeated measures of HbA1c or glycosylated haemoglobin. Title and abstract screening was performed in duplicate, full text screening and data extraction by one reviewer and verified by a second. Risk of bias of included papers was assessed using a modified consensus-based standards for the selection of health measurement Instruments (COSMIN) tool. Intraclass correlation coefficient (ICC) results were pooled with a meta-analysis and coefficient of variation (CV) results were described by median and range.

**Results:**

Of 2675 studies identified, 111 met the inclusion criteria. Twenty-five studies reported variability data in healthy patients, 19 in patients with type 1 diabetes and 59 in patients with type 2 diabetes. Median within-subject coefficient of variation (CV) was 0.070 (IQR 0.034 to .09). For healthy subjects the median CV for HbA1c % was 0.017 (IQR 0.013 to 0.022), for patients with type 1 diabetes 0.084 (IQR 0.067 to 0.89) and for type 2 diabetes 0.083 (IQR 0.06 to 0.10). CV increased with mean population HbA1c.

**Limitations:**

Assessment of variability was not the main aim of many of the included studies and some relevant papers may have been missed. Many included papers had few participants or few repeated measurements.

**Conclusions:**

Within-subject variability of HbA1c is higher for patients with than without diabetes and increases with mean population HbA1c. This may confound observed relationships between HbA1c variability and health outcomes. Because of its importance in clinical decision-making there is a need for better estimates and understanding of factors associated with of HbA1c variability.

## Introduction

Haemoglobin A1c (HbA1c) is produced by non-enzymatic glycation of haemoglobin. It provides an estimate of glycaemia (mean glucose levels) over the preceding one to three months, proportionately weighted to more recent periods [1]. It is therefore used to diagnose diabetes mellitus and to monitor patients with diabetes. The American Diabetes Association (ADA) recommends a cut-off point of more than or equal to 6.5% (48mmol/mol) HbA1c level for a diagnosis of diabetes mellitus and 5.7–6.4% (39–47 mmol/mol) for a diagnosis of prediabetes [2]. It is recommended that HbA1c is measured every three to six months in newly diagnosed patients with type diabetes mellitus until stabilised, and thereafter every six months [3].

HbA1c levels can be reported using either International Federation of Clinical Chemistry and Laboratory Medicine (IFCC), expressed in mmol/mol, or National Glycohemoglobin Standardization Program (NGSP) reference systems, expressed as % [4]. Although a conversion formula between the two methods is available, they may not be directly interchangeable [5]. Further, within-subject variability in NGSP units is reported to be lower than in IFCC units [6]. Although the IFCC is considered to be the higher standard test, many countries continue to use NGSP units [7].

Reference ranges for diagnostic tests are usually set by comparison to a reference population, which allows assessment of between-subject variation. However, biological parameters also vary over time. This can be systematic and predictable, such as seasonal variation [8], or may be due to chance. This longitudinal within-subject variation is known as biological variation. Variation can also be introduced into a measurement from pre-analytical factors such as stress, exercise and food intake prior to the laboratory measurement and posture during the sampling procedure. Imperfect accuracy and precision of laboratory measurement mean that if measurement is repeated a number of times on the same sample, there will be a range of results around the true value. This is analytical variation.

The variability in measured HbA1c results encountered in real world clinical practice is because of a combination of biological, pre-analytical and analytical variability. Biological variability is the greatest component of total variability, since clinical laboratories usually set acceptable analytical variability at <0.5% of biological variability [9].

The greater the within-subject variability of a parameter, the lower the probability that a single measurement is an accurate reflection of the true mean of the parameter in that individual. This can lead to errors in diagnosis, prognosis and treatment. Specifically for HbA1c, an inaccurate result may lead to an incorrect, missed or delayed diagnosis of diabetes mellitus, causing inappropriate commencement of treatment or an inappropriate delay in commencing treatment, and an incorrect estimation of glycaemic control in patients with diabetes, leading to an inappropriate increase in treatment intensity or a delay in intensifying treatment.

Three previous systematic reviews on HbA1c variability have been published. Gonzalez-Lao et al [10] reported the results of a systematic review and meta-analysis of 17 papers. Based on three papers this gave a pooled estimate of within-subject coefficient of variation (CV_i_) for HbA1c in healthy adults of 0.013 (0.012–0.025) for results reported in IFCC units (mmol/mol) and based on four papers, a CV_i_ of 0.013 (0.012–0.021) for results reported in non-IFCC units (%). CV_i_ was slightly higher in patients with diabetes mellitus than subjects without diabetes. The European Federation of Clinical Chemistry and Laboratory Medicine (EFLM) database reports a CV_i_ of 0.016 (0.013–0.024) for papers reporting IFCC units based on four papers and 0.012 (0.003–0.019) for papers reporting NGSP units (based on 7 papers) [11]. Braga et al performed a systematic review without meta-analysis of eight studies, with CV_i_s ranging from <0.007 to 0.98 [12].

There is a need for an up to date and more comprehensive systematic review of within-subject variability of HbA1c.

### Aim of review

To describe and evaluate the current literature on within-subject variability of measured HbA1c and from this to estimate variability in people with and without diabetes mellitus.

## Methods

Searches were devised to identify cohort studies, clinical trials or any studies in which an HbA1c or glycosylated haemoglobin measurement was performed more than once in the same individual. See Appendix 1 in S1 File for sample Medline and Embase search strategy. The searches for this study were combined with a similar study into C-reactive protein (CRP) variability, and then CRP studies were excluded at the full text screening stage.

The international prospective register of systematic reviews, PROSPERO, was checked for ongoing reviews, and the protocol was registered with PROSPERO. On 5^th^ August 2020, database searches were carried out. Medline, Embase, Cochrane Central, Epistemonikos and Open Grey were searched from inception to 5^th^ August 2020. An update to the search was performed to include studies published up to November 2022 (Full details in Appendix 2 in S1 File). Subject experts were contacted for suggestions for further papers. Search terms were adapted for each database searched. The references of included papers were checked by hand for further relevant papers. Endnote reference management software was used to collate studies revealed by the search.

Studies were included if primary research data on the variability of at least two measurements of HbA1c or glycosylated haemoglobin within the same subject was recorded. Studies could be of any design. The population included adults and children, healthy or with any disease condition, in any setting. Outcome was variability which could be reported as coefficient of variation (CV_I_), standard deviation (SD), variability independent of the mean (VIM), index of individuality (II), Reference Change Value (RCV), index of heterogeneity, validity coefficient (VC), ICC agreement, ICC consistency, Cronbach’s alpha or Cohen’s kappa. There was no restriction on time of publication, language of publication, population, setting or sample size. Studies were excluded if participants were not in a steady state (measurements were before and after an intervention or had an acute or rapidly changing illness) or data were secondary (systematic and narrative reviews).

Studies were grouped for synthesis based on variability measure reported eg ICC, CV. Since this study was aimed to capture variability data wherever it was published, there was no limit on publication date, language or study design.

Titles and abstracts were screened independently by two reviewers (AG and EF) in Abstrackr systematic review software [13], and those identified of interest underwent full text searches. Full texts were screened by AG and exclusions confirmed by EF. Differences were resolved by discussion. Foreign language papers were translated by Google translate software. Data were extracted into Excel from the full text of the study papers where possible, and from abstracts where full texts were not available, by a single reviewer (AG). All data extraction was verified independently by a second reviewer (EF). Where the same study was reported in multiple papers, the full text paper was preferred over an abstract, English language preferred over non-English, and the earliest English version over the later if there was more than one.

Table 1 lists the outcome and other main variables extracted. All eligible outcomes were included where more than one outcome was reported within a paper. The primary outcome measure was variability of repeated measurements within the same subject. Where multiple measures of variability were given in a single study, the primary population only was analysed for the main meta-analysis. The primary population was the full study population (as opposed to subgroups), and if the full study population was not given, the primary outcome was identified using the following hierarchy: 1. Healthy population; 2. Most stable population (i.e. subjectively judged to be in the most steady-state such as disease course or treatment); 3. First outcome listed in the paper.

**Table 1:** Variables extracted from papers.

Variable	Definition and rules
Study design	Cohort, RCT etc
Number of subjects	Number of subjects used to calculate variability measure
Age	Average age of subjects used to calculate variability measure. If the subjects used to calculate variability measure is a subset of the study population and only the age is given for the whole population, the age of the whole population is used.
Sex	Percentage of subjects used to calculate variability measure who were male. If the subjects used to calculate variability measure is a subset of the study population and only the sex is given for the whole population, the sex of the whole population is used.
Ethnicity	Ethnicity recorded yes/no? If country of origin of subjects only is recorded, this is counted as no.
Setting	Primary care/community versus secondary/tertiary/laboratory setting
Health status	Healthy, type I diabetes mellitus, type II diabetes mellitus or other
Number of measurements	Number of repeated HbA1c measurements in the same individual
Maximum time interval	Maximum time interval between consecutive measurements
Minimum time interval	Minimum time interval between consecutive measurements
Method of variability calculation	Is the variability calculation described?
Unit of measurement	Mmol/mol or %
Variability described as total or component parts?	Is variability calculation explicitly described as total variability, within-subject variability, or uncertain?
CV_i_	Coefficient of variation of repeated measures within an individual
SD	Standard deviation of repeated measures within an individual
ICC	Intraclass correlation of repeated measures within an individual

Where information was missing or unclear, the information was not extracted with the following exception: where patient characteristic data was reported only for a whole study population, but variability data was only present for a sub-population, the patient characteristic data for the whole population was used.

A risk of bias tool was used adapted from the Consensus-based Standards for the selection of health Measurement Instruments (COSMIN) risk of bias tool for test reliability [14]. The template risk of bias form with associated explanatory notes is shown in Appendix 2 in S1 File. Risk of bias was assessed by AG and reviewed by EF. Disagreements between reviewers were resolved by discussion.

The main outcome measure of this review is coefficient of variation of repeated measurements within the same individual.

All studies were considered for synthesis provided they had a measure of variability that had a sufficient number of studies reported so they could be pooled. Coefficients of variation were converted from % to a decimal fraction.

Stata SE 17 was used to perform statistical calculations and generate forest plots. ICCs were transformed using Fisher’s Z transformation [15]. Z scores were then back-transformed (see Appendix 4 in S1 File) and 95% confidence intervals of the ICC were calculated. Coefficient of variations were considered for meta-analysis, but no well-described and validated methods currently exist for meta-analysis of coefficient of variations, so we describe the results identified here. Descriptive subgroup analyses were performed based on unit of measurement (IFCC or NGSP), health status, setting, number of measurements and whether short or long-term variability was measured. Sensitivity analyses were based on number of subjects and risk of bias.

## Results

(Fig 1).

**Fig 1:**
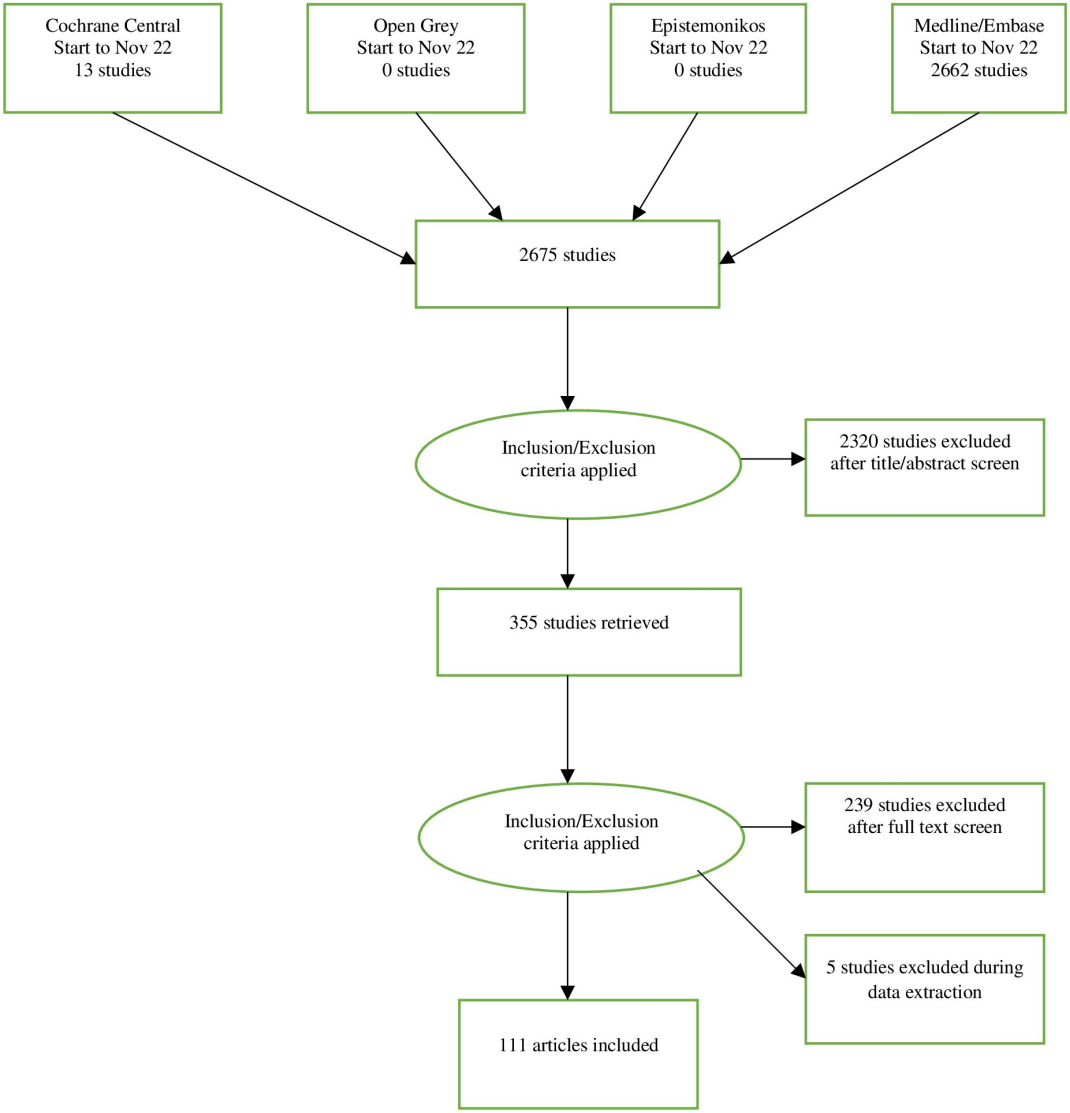
PRISMA flow diagram describing selection of studies.

After database searches and hand searching of reference lists of included studies, 2,675 non-duplicate studies were identified of which 2320 were excluded after title and abstract screening leaving 355 studies. After full text screening, 244 were excluded, and a further five during data extraction. Most full text exclusions were because no recognised measure of variability was reported.

One hundred and eleven studies met the inclusion criteria (31.3% of full text studies screened). Sample sizes ranged from 4 to 91,866 subjects, with a median of 378. One hundred-and-five studies (95%) reported longer term variability (arbitrarily defined for this study as seven or more days between measurements), two reported short-term variability (less than seven days between measurements), one reported both long and short term, and three did not report measurement intervals. One hundred and six studies (95.5% of included studies) were of a cohort design, three (2.7%) used data from the placebo arm of a randomised controlled trial and two (1.8%) were case-control studies Twenty studies (18.0%) were carried out in a primary care or community setting and 73 (65.8%) studies were carried out in secondary or tertiary setting such as universities, hospitals and laboratories. In eighteen (16.2%) studies the setting was not reported.

Study populations were diverse in terms of age, gender and health status. Ethnicity was recorded in 34 (30.6%) studies. Included studies and full study characteristics are presented in Appendix 4 in S1 File. With questions four and five of the risk of bias scoring excluded, 21 (18.9%) studies scored the best risk of bias score of one, 53 (47.7%) studies scored a risk of bias of two, and 37 (33.3%) studies scored a risk of bias of three. Risk of bias scores are presented in Appendix 5 in S1 File. Table 2 summarises the study characteristics of the included papers.

**Table 2:** Summary statistics of study characteristics of included papers.

Study characteristic	Number of studies reporting the characteristic (out of 111)	Median value of characteristic	Lower quartile	Upper quartile	Minimum	Maximum
Number of subjects	110	473.5	59	2103	4	91866
Age	84	59.05	44	64.79	8.77	76
% male	88	52.34048	46.485	58.11	0	98
Number of measurements	98	5	4	10.4	2	36.8

Included studies consisted of ninety-four full texts and seventeen abstracts. The average study age of subjects in the primary population ranged from 8.8 to 76 years, with a median of 59.1. The percentage of subjects who were male ranged from 0% to 98% with a median of 52.3%. Ninety-three studies reported the setting. Twenty of the 93 (21.5%) studies that recorded setting were conducted in primary care or the community and 73/93 (78.5%) in secondary or tertiary care or in a laboratory/university situation. Health status of individuals was recorded in 110/111 studies of which 25 (22.5%) were healthy, 59 (53.2%) had type 2 diabetes, 19 (17.1%) had type 1 diabetes, 4 (3.6%) had both type 1 diabetes and type 2 diabetes, and 3 (2.7%) had other diseases.

The average number of measurements ranged from 2 to 36.8 with a median of 5. Eighty-four (75.7%) studies used NGSP (%) units to calculate the variability and four (3.6%) used IFCC (mmol/mol); 23 (20.7%) reported in both units or did not clearly specify the units.

Eighty-three (74.8%) studies reported total variation, 15 studies (13.5%) reported individual variation, and 1 (0.9%) study reported both. For 12 (10.8%) studies the type of variation reported was unclear). The CV_I_ of the HbA1c primary population ranged from 0.0029 to 0.179, with a median of 0.070 (IQR 0.051). The within-subject standard deviation of the HbA1c of the primary population where the units were NGSP (%) ranged from 0.08 to 1.47 with a median of 0.62 (IQR 0.37). Only five papers reported ICC, with a median of 0.873 and a range of 0.59 to 0.97.

One paper [16] appears to report an incorrect value for the CV since the value appears to be implausibly low compared to other reports of this result implausible and is also inconsistent with the other variability measures reported in the paper. The value for CV from this paper used in this review was therefore calculated from the reported SD and mean. Two papers did not report a CV, ICC or SD. Segar [17] reported variability as average successive variability (average absolute difference between successive values), with a result of a mean of 0.6±0.3% (mean±SD). Sugawara [18] used an adjusted standard deviation to account for different numbers of HbA1c measurements, reporting an adjusted SD result of 0.79±0.6% (mean±SD). Three papers [19–21] reported variability measurements for units of both mmol/mol and % and the % unit is included in this review.

Median within-subject coefficient of variation (CV_i_) was 0.070 (IQR 0.034 to .09). Table 3 summarises the results of variability measures of the primary study population.

**Table 3:** Summary of results of variability measures of primary study population. (See Methods for definition).

Study result	Number of studies reporting result (out of 111)	Median value of result	Lower quartile	Upper quartile	Minimum	Maximum
CV_i_	89	.0698	.0392	.0904	.0029	.179
ICC	6	.8115	.74	.93	.59	.97
SD (% units)	35	0.62	0.4	0.77	0.080	1.47

**Fig 2:**
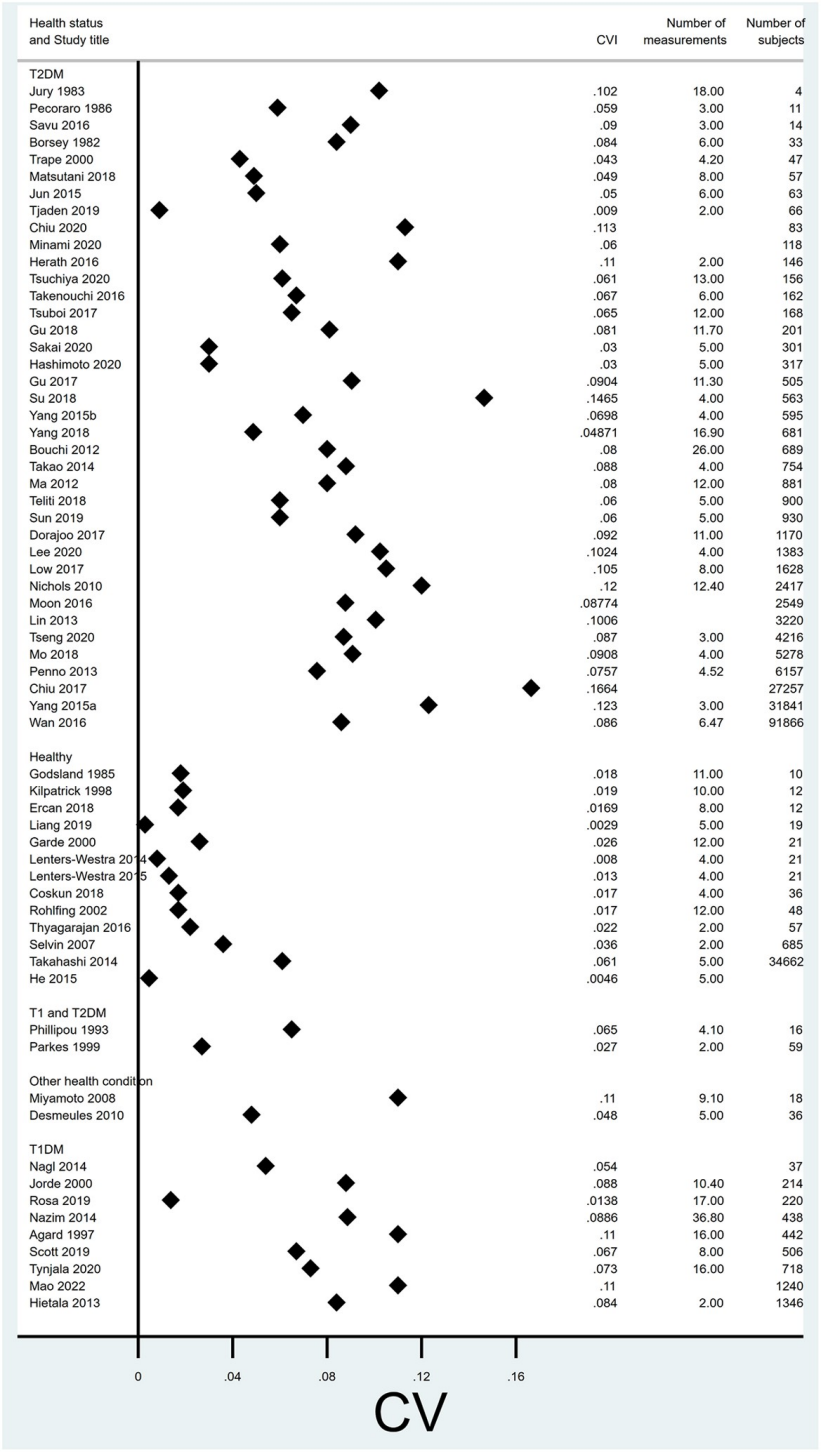
Forest plot of CV for studies reporting in units of %, split by health status.

**Fig 3:**
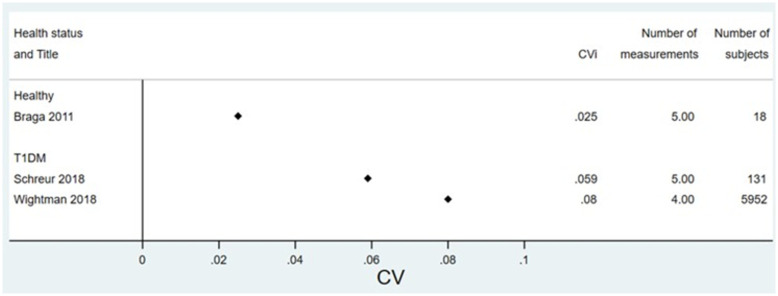
Forest plot of CV for studies reporting in units of mmol/mol, split by health status.

With units reported as %, CV_i_ in healthy subjects (median 0.017; IQ range: 0.013 to 0.022) was lower than in patients with type 2 diabetes (0.083; IQ range: 0.06 to 0.10), type 1 diabetes (0.084; IQ range 0.067 to 0.89) or other health conditions (0.079; IQ range 0.048 to 0.11) (Fig 2 and Table 4). Findings were similar for mmol/mol units (Fig 3).

**Table 4:** Summary statistics of reported CV_I_ reporting in units of %, split by health status.

Health status	Observations	Median CV_i_	Lower quartile	Upper quartile	Min	Max
Healthy	13	0.017	0.013	0.022	0.0029	0.061
Type 2 diabetes	38	0.083	0.06	0.10	0.009	0.166
Type 1 diabetes	9	.084	.067	.0886	.0138	.11
Types 1 and 2 diabetes	2	0.046	0.027	0.065	0.027	0.065
Other	2	0.079	0.048	0.11	0.048	0.11

**Fig 4:**
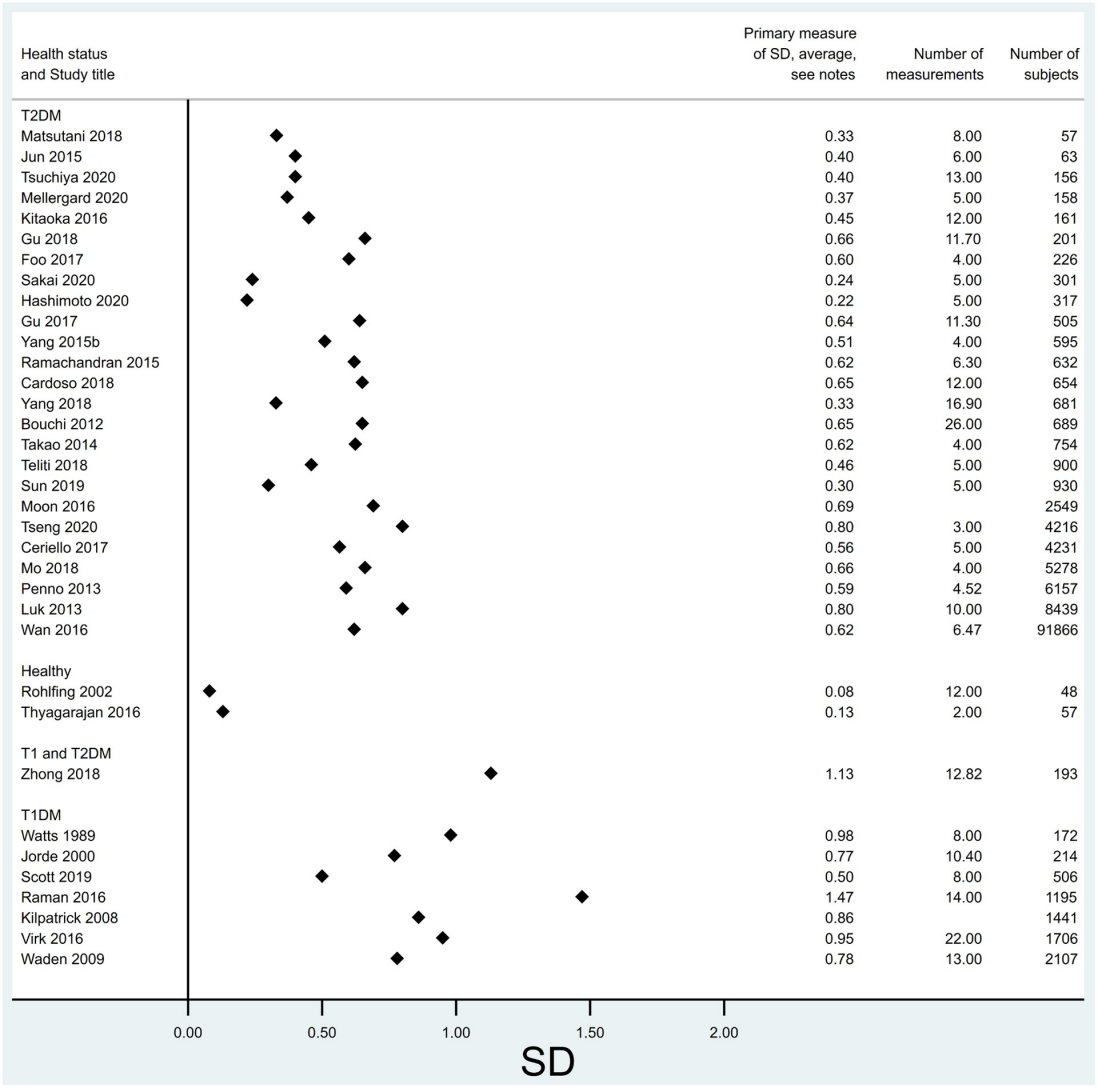
Forest plot of SD for studies reporting in units of %, split by health status. T2DM = type 2 diabetes mellitus, T1DM = type 1 diabetes mellitus.

**Fig 5:**
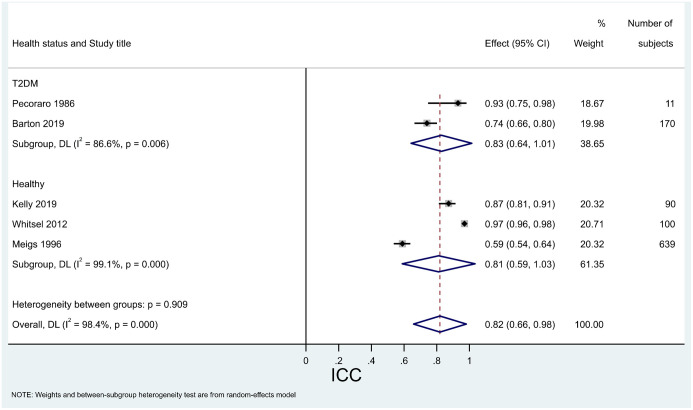
Forest plot with meta-analysis of ICC for studies reporting units of %, split by health status. T2DM = type 2 diabetes mellitus.

Figs 4 and 5 show results for studies measuring variability as SD or ICC.

Forest plots for CV for studies reporting units of %, stratified by risk of bias, setting, short- or long-term measurements and whether CVI or CVT was reported or whether this was uncertain can be found in Appendix 7 in S1 File.

**Fig 6:**
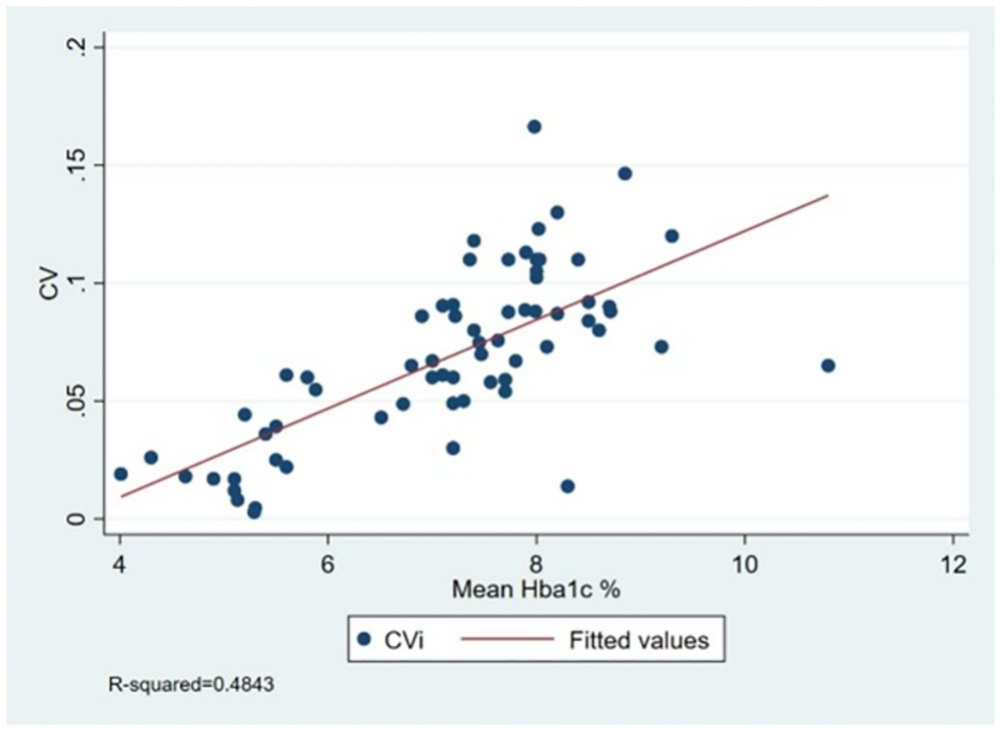
Scatter plot of mean population HbA1c against CV, with line of best fit.

Because of the variability was higher in patients with diabetes, for all studies where it was reported we plotted mean population HbA1c against CV_i_ (Fig 6). These demonstrated a positive correlation (R^2^ 0.48).

## Discussion

This review is the most comprehensive systematic review and meta-analysis of within-subject HbA1c variability. Of the three previous systematic reviews, one did not perform a meta-analysis and two only performed meta-analyses on healthy populations. The largest included only 17 studies in total, 3 the meta-analysis of variability measured using of mmol/mol and 4 in the meta-analysis of % units [10]. This review included 111 studies. Previous systematic reviews on HbA1c variability were designed to estimate a coefficient of variation under ideal conditions in order to inform analytical performance specifications, reference change values and population-based reference intervals. By contrast the current study was designed to be as broad as possible, increasing generalisability by including HbA1c variability in a variety of settings and health conditions.

There is no well-described, validated method for meta-analysis of CV_i_s therefore it was not undertaken in this study.

Our median estimates of CV_i_ (NGSP %) were 0.017 (IQ range: 0.013 to 0.022) in healthy individuals, 0.083 (IQ range: 0.06 to 0.10) in type 2 diabetes and 0.084 (IQ range 0.067 to 0.89) in type 1 diabetes. These are similar to previous reviews of healthy patients which report a median CV of 0.013 (95% CI 0.012–0.025) for IFCC (mmol/mol) units and 0.013 (0.12–0.21) for NGSP(%) [10]; 0.016 (95%CI 0.013–0.024) for IFCC(mmol/mol) and NGSP (%) 0.011 (0.002–0.019) for % [11]. This is consistent with previous research suggesting that patients with diabetes have a higher CV_I_ of HbA1c than healthy patients [10, 22].

We demonstrated a correlation between mean population HbA1c and within-subject variation measured by CV_i_. This suggests subjects with prediabetes have a higher HbA1c variability than the healthy and poorly-controlled patients with diabetes have a higher variability than well-controlled patients with diabetes. This may be due to different levels of insulin resistance, more than variation in caloric intake. It also means clinical outcomes which correlate with mean HbA1c will also correlate with CV_i_. Researchers investigating variability as a predictor of outcomes should consider using a measure of variability which is independent of the mean.

The variability of HbA1c has implications for clinical decision making. Misclassification of type 2 diabetes may be considered a low risk since the HbA1c within-subject variability is lower, although with borderline results, a single reading may still be above the cutoff for a diagnosis of type 2 diabetes when the true mean is below the cutoff point. General practitioners’ understanding of this issue derived from clinical experience may explain why they often do not commence treatment with oral anti-diabetic drugs until they document a HbA1c reading well above the cutoff recommended by National Institute for Healthcare and excellence (NICE) guidelines [23]. Of even more importance are the implications for monitoring and treatment decisions, since the variability is higher in these patients. Metformin monotherapy lowers HbA1c by 1.1% NGSP units (12mmol/mol IFCC units on average) [24] which is within the usual range of within-subject variation. This is consistent with the finding that 15–18% of patients that commence treatment with oral anti-diabetic drugs demonstrate an increase in measured HbA1c –this can be explained by within-subject variation.

Another concern is that higher HbA1c variability is associated with an increased all-cause mortality due to a variety of mechanisms such as endothelial dysfunction, increased oxidative stress and increased release of cytokines [25], demonstrating the importance of reducing HbA1c variability by improving control of diabetes. Moreover, a strict glycemic control may play a cardioprotective effect [26].

Two obvious outliers were found in this review, one which showed a CV_i_ of 0.59 [16]. This appeared to be an error and the CV_i_ was recalculated for this review from data available in the study to give a result of 0.086. The other study showed a CV_i_ of 0.0029 [27], although there is an error in the abstract where a different CV_i_ result is given, which appears to be the result for males only. The authors acknowledge their result is lower than previously published estimates of HbA1c CV_I_ and suggest this may be due to strict control of pre-analytic factors and Chinese ethnicity of the subjects. This latter seems unlikely since four other papers with Chinese subjects reported a CV_I_ ranging from 0.075 to 0.17 [16, 28–30]. Nevertheless, it has been shown that ethnicity influences HbA1c levels [31]. This does illustrate the importance of taking ethnicity into account when studying variability, and only 34/111 studies in this review explicitly reported the ethnicity of their subjects, although it is not always clear whether nationality or ethnicity is being referred to. Future research in HbA1c variability should include the ethnicity of subjects.

There are a number of weaknesses with some of the studies in this review. Because variability was not the primary outcome of the studies some relevant studies may not have been identified. Some included studies had low numbers of subjects and/or low numbers of repeat measurements. It was not always clear whether studies were reporting total variability (i.e. CV_A_ + CV_i_) or within-subject variability (CV_i_). S3 Fig in Appendix 7 of S1 File illustrates the CVs of studies split by whether they reported CV_I_, CV_T_, or whether this was uncertain. Subjectively there seemed to be minimal difference between the groups.

HbA1c may not be an ideal measure of glycaemic control because it does not capture hypoglycaemic events. In the future it may be replaced by flash glucose monitoring for some patients, but at present it is the main parameter used to assess glycaemic control and is likely to remain so for most patients.

Twenty-three studies did not report the method with which they calculated variability. This is particularly important with ICCs since there are at least ten different ways of calculating ICC which can all give different results [32].

Most studies reported results in units of % (NGSP method) despite mmol/mol (IFCC method) being recommended. In some studies, it was unclear whether results were expressed using NGSP or IFCC methods, which is important since these methods can give different results.

A bespoke risk of bias tool was developed and included in the original protocol recorded in Prospero, but it was ultimately decided preferable to use a validated risk of bias tool, and the COSMIN tool was selected. This has some limitations, particularly when it comes to the questions regarding blinding. Generally, in studies into biological variability the investigators are not blinded to previous measurements. These questions were therefore omitted when a study’s risk of bias score was assigned. However, this is unlikely to have affected findings as HbA1c is measured objectively.

Risk of bias due to missing publications was deemed to be low, since in most cases the outcome measure of this review was not the primary outcome of the individual studies, and therefore likelihood of publication is unlikely to be related to variability outcomes. Some papers with repeated measures do not report variability data, but this is unlikely to be related to variability outcomes.

As some publications included multiple populations, with information being given only on the subgroups and not the total, arbitrary decisions had to be made as to which populations to include in the primary analysis. However, the rules to decide which group to include were applied systematically, using the following hierarchy: 1. Healthiest population; 2. Most stable population; 3. First listed population. Some studies reported demographic data for the whole population but not for the subgroup for which variability data was reported. In these cases, the demographic data for the whole study population was used and was assumed to be close to the variability subgroup. Although a larger number of studies has been included than in previous reports, the quality of the studies and the other limitations of the review mean that an accurate real-world estimate of within-subject variability of HbA1c remains lacking. Further studies with larger numbers of subjects with a variety of health conditions is important to understand the extent to which HbA1c varies within a subject over time in a clinical setting. This would help inform clinicians when making diagnostic and treatment decisions, and the authors of clinical guidelines when making recommendations on diagnosis and treatment.

## Conclusion

This comprehensive systematic review of estimates of HbA1c variability includes data from 111 papers. We provide separate estimates of HbA1c variability in healthy and diabetic populations. We also observe a positive correlation between mean HbA1c and within-subject variability.

## Supporting information

S1 File (DOCX)

S1 Checklist (DOCX)

S1 Data (XLSX)

## Abbreviations

COSMIN: Consensus-based standards for the selection of health measurement Instruments
CRP: C-reactive protein
CV_A_: analytical coefficient of variation
CV_I_: within-subject coefficient of variation
EFLM: European Federation of Clinical Chemistry and Laboratory Medicine
Hba1c: Haemoglobin A1c
IFCC: International Federation of Clinical Chemistry and Laboratory Medicine
NGSP: National Glycohemoglobin Standardization Program
