# Supplementary material for: Within-subject variation of HbA1c: A systematic review and meta-analysis
Source: PLoS One. 2023 Aug 2;18(8):e0289085. doi: 10.1371/journal.pone.0289085 (PMC10395823; doi:10.1371/journal.pone.0289085)
Supplement: S1 File — (DOCX) [file pone.0289085.s001.docx]

# Supplementary Materials

# Appendix 1

Search strategies

#### (Medline, Embase, Medline and In-Process, In-Data-Review & Other non-indexed citations)

Sample search strategy, Medline 1946-Nov 2022 and In-Process and other non-indexed citations

1. (CRP or c-reactive protein).mp. [mp=title, abstract, original title, name of substance word, subject heading word, floating sub-heading word, keyword heading word, organism supplementary concept word, protocol supplementary concept word, rare disease supplementary concept word, unique identifier, synonyms]

2. Glycated Hemoglobin A/ or HbA1c.mp.

3. biological varia$.mp. [mp=title, abstract, original title, name of substance word, subject heading word, floating sub-heading word, keyword heading word, organism supplementary concept word, protocol supplementary concept word, rare disease supplementary concept word, unique identifier, synonyms]

4. within-individual varia$.mp.

5. within-subject varia$.mp. [mp=title, abstract, original title, name of substance word, subject heading word, floating sub-heading word, keyword heading word, organism supplementary concept word, protocol supplementary concept word, rare disease supplementary concept word, unique identifier, synonyms]

6. within-individual CV.mp. [mp=title, abstract, original title, name of substance word, subject heading word, floating sub-heading word, keyword heading word, organism supplementary concept word, protocol supplementary concept word, rare disease supplementary concept word, unique identifier, synonyms]

7. Within-subject CV.mp. [mp=title, abstract, original title, name of substance word, subject heading word, floating sub-heading word, keyword heading word, organism supplementary concept word, protocol supplementary concept word, rare disease supplementary concept word, unique identifier, synonyms]

8. Intraindividual CV.mp. [mp=title, abstract, original title, name of substance word, subject heading word, floating sub-heading word, keyword heading word, organism supplementary concept word, protocol supplementary concept word, rare disease supplementary concept word, unique identifier, synonyms]

9. intra-individual varia$.mp.

10. day-to-day.mp.

11. visit-to-visit.mp.

12. Coefficient of variation.mp.

13. (cvi or cvw or cvb or cvwithin-subject or cvbiological).mp. [mp=title, abstract, original title, name of substance word, subject heading word, floating sub-heading word, keyword heading word, organism supplementary concept word, protocol supplementary concept word, rare disease supplementary concept word, unique identifier, synonyms]

14. chronic venous insufficiency.mp.

15. vaccine immunology.mp.

16. content validity index.mp.

17. cerebral visual impairment.mp.

18. community virtual ward.mp.

19. cardiac vagal withdrawal.mp.

20. coronary vessel wall.mp.

21. variability independent of the mean.mp.

22. VIM.mp.

23. thalamus.mp. or Thalamus/

24. lactamase.mp. or beta-Lactamases/ or beta-Lactamase Inhibitors/

25. index of individuality.mp.

26. Reference Change Value.mp.

27. rcv.mp.

28. (intraclass or ICC).mp. [mp=title, abstract, original title, name of substance word, subject heading word, floating sub-heading word, keyword heading word, organism supplementary concept word, protocol supplementary concept word, rare disease supplementary concept word, unique identifier, synonyms]

29. Revue canadienne du vieillissement.mp.

30. recoverin.mp. or Recoverin/

31. index of heterogeneity.mp.

32. validity coefficient.mp.

33. reliability parameter.mp.

34. (animal not human).mp. [mp=title, abstract, original title, name of substance word, subject heading word, floating sub-heading word, keyword heading word, organism supplementary concept word, protocol supplementary concept word, rare disease supplementary concept word, unique identifier, synonyms]

35. 1 or 2

36. 3 or 4 or 5 or 6 or 7 or 8 or 9 or 10 or 11 or 12 or 13 or 25 or 26 or 28 or 31 or 32 or 33

37. 14 or 15 or 16 or 17 or 18 or 19 or 20 or 23 or 24 or 29 or 30

38. 35 and 36

39. 38 not 37

40. 39 not 34

#### B. Cochrane central

(CRP) or (HbA1c)

AND

variability or variation or within-individual or within-subject or day-to-day or test-retest

#### C. Epistemonikos

(title:((CRP or HbA1c) AND variability) OR abstract:((CRP or HbA1c) AND variability))

D. Open Grey

(CRP or HbA1c) and variability

# Appendix 2

**S1 table 1. Databases searched**

| Database  Ovid  Medline  Embase  Medline and In-Process, In-Data-Review & Other non-indexed citations  Cochrane Central  Epistemonikos  Open Grey | Coverage  1946 to 11/22  1974 to 11/22  1946 to 11/22  Inception to 11/22  Inception to 11/22  Inception to 11/22 |
| --- | --- |

# Appendix 3

## Risk of bias template and explanatory notes

Domains assessed were divided into two groups. Group A addressed elements of the research question, which included the name of the instrument, the measurement property (variability measure), and the source of the variation and the patient population. Group B scored risk of bias using 7 questions: were the patients stable?; was the time between measurements appropriate?; were the measurements similar for the repeated measurements except for the source of the variation (in this case time)?; was the measurement administered without knowledge of previous scores in the patient?; were scores assigned without knowledge of previous scores in the patient?; were there any other important flaws in the design or statistical methods?; was a recognised measure of variability calculated with the method of calculation shown? Each of the 7 questions of group B were scored with the following system: 1=very good; 2=adequate; 3=doubtful; 4=inadequate.

**S1 table 2: Risk of Bias form, Part A: Elements of Research Question**

| Element | Instruction | Result |
| --- | --- | --- |
| 1. Name of the instrument | CRP, hsCRP or HbA1c |  |
| 2. Version or way of operationalization | Method of analysis |  |
| 3. Construct | Description of what is being measured |  |
| 4. Measurement property. | Variability measure(s) |  |
| 5. Components that will be repeated | Whole measurement? Parts? | Whole |
| 6. Source(s) of variation varied | Component which is varied across the measurements (time?) Unintentional sources of variation | Time |
| 7. Patient population |  |  |
|  |  |  |

Notes:

4. Reliability study (relative eg ICC) or measurement error eg SD, CV, and which measurement

6. Any deficiencies that may introduce unintentional sources of variation eg training, preparation, collection, transport, test methodology

**S1 table 3: Risk of Bias form, Part B: Risk of bias rating**

| Design requirements | Rating* | Reason/evidence |
| --- | --- | --- |
| 1. Were patients stable in the time between the repeated measurements on the construct to be measured? |  |  |
| 2. Was the time interval between the repeated measurements appropriate? |  |  |
| 3. Were the measurement conditions similar for the repeated measurements – except for the condition being evaluated as a source of variation? |  |  |
| 4. Did the professional(s) administer the measurement without knowledge of scores or values of other repeated measurement(s) in the same patients? |  |  |
| 5. Did the professional(s) assign scores or determine values without knowledge of the scores or values of other repeated measurement(s) in the same patients? |  |  |
| 6. Were there any other important flaws in the design or statistical methods of the study? |  |  |
| 7. Was the Coefficient of Variation (CV), ICC, RCV, SD, VIM, variance, ASV, II calculated? |  |  |

* Very good/adequate/doubtful/inadequate/NA

Paper notes:

Guidance Notes:

Ratings are as follows unless described otherwise below:

Very good: yes; Adequate: reasons to assume standards were met; Doubtful: unclear; Inadequate (no)

1. Completely stable is very good, long term illness is adequate, short term/acute illness is doubtful, unstable should have been excluded at screening

2. Any time interval is allowed for the purposes of this review

3. Environmental change may count as doubtful

6. Very good: no; Doubtful: minor methodological flaws; inadequate: yes

7. Very good: the model or formula for the variability measure is described; it matches the reviewer constructed research question and the data;

Adequate: the model or formula is not described or does not optimally match the reviewer constructed research question and evidence provided that no systematic difference has occurred

Doubtful: no knowledge about systematic difference or with evidence provided that systematic difference has occurred

Inadequate: variability calculated based on Cronbach’s alpha, or using SD from another population

# Appendix 4

**S1 table 4:** **Included papers and characteristics**

| Paper | Number of subjects | Age (primary analysis) | % male (primary analysis) |
| --- | --- | --- | --- |
| Agard 1997 [33] | 442 | 35 | 53.17 |
| Barton 2019 [34] | 170 |  | 0.00 |
| Borsey 1982 [35] | 33 | 52 | 89.29 |
| Bouchi 2012 [36] | 689 | 65 | 57.18 |
| Braga 2011 [37] | 18 |  | 50.00 |
| Cardoso 2018 [38] | 654 | 60.1 | 38.10 |
| Carlsen 2011 [39] | 15 |  | 40.00 |
| Ceriello 2017 [40] | 4231 | 67.4 | 50.90 |
| Chiu 2020 [28] | 83 | 64.5 | 59.04 |
| Chiu 2017 [41] | 27257 |  | 46.77 |
| Coskun 2018 [42] | 36 |  | 52.78 |
| Critchley 2019 [43] | 58832 | 67.7 | 55.30 |
| Dergez 2014 [44] | 20 |  |  |
| Desmeules 2010 [45] | 36 | 14 | 66.67 |
| Dorajoo 2017 [46] | 1170 | 56.1 | 52.39 |
| Ercan 2015 [47] | 15 |  |  |
| Ercan 2018 [48] | 12 | 43.5 | 75.00 |
| Foo 2017 [49] | 226 | 62 | 87.70 |
| Garde 2000 [50] | 21 |  | 0.00 |
| Godsland 1985 [51] | 10 |  | 60.00 |
| Gu 2018 [52] | 201 | 65.2 | 60.20 |
| Gu 2017 [53] | 505 | 69.6 | 52.08 |
| Hashimoto 2020 [54] | 317 | 66.7 | 56.78 |
| He 2015 [55] | NR |  |  |
| Herath 2016 [56] | 146 |  |  |
| Hermann 2014 [57] | 35891 | 16.2 | 52.30 |
| Hietala 2013 [58] | 1346 | 38.7 | 52.08 |
| Hirakawa 2014 [59] | 4399 | 65.5 | 42.60 |
| Howey 1989 [60] | 8 |  | 37.50 |
| Hsu 2012 [61] | 821 |  | 46.10 |
| Jang 2019 [62] | 6462 | 50 | 50.00 |
| Jorde 2000 [63] | 214 | 30.4 | 62.15 |
| Jun 2015 [64] | 63 | 59.5 | 63.49 |
| Jury 1983 [65] | 4 |  |  |
| Kelly 2019 [66] | 90 | 52.8 | 31.00 |
| Kilpatrick 1998 [67] | 12 | 40 | 58.33 |
| Kilpatrick 2008 [68] | 1441 | 27 | 52.80 |
| Kitaoka 2016 [69] | 161 | 62.6 | 47.50 |
| Lee 2020 [70] | 1383 | 65.6 | 52.00 |
| Lenters-Westra 2015 [6] | 21 |  |  |
| Lenters-Westra 2014 [71] | 21 |  | 52.38 |
| Li 2017 [72] | 15875 | 69 | 45.12 |
| Liang 2019 [27] | 19 | 26 | 47.37 |
| Lin 2013 [73] | 3220 | 57.78 | 48.91 |
| Low 2017 [74] | 1628 |  | 57.90 |
| Lui 2020 [29] | 83282 | 71.3 | 46.20 |
| Luk 2013 [75] | 8439 | 57 | 47.00 |
| Ma 2012 [76] | 881 | 60 | 47.90 |
| Mao 2022 [77] | 1240 | 49 | 52.20 |
| Matsutani 2018 [78] | 57 | 67.2 | 68.42 |
| Meigs 1996 [79] | 639 | 73.6 | 35.80 |
| Mellergard 2020 [19] | 158 | 67.6 | 65.20 |
| Minami 2020 [80] | 118 | 74.1 | 58.47 |
| Miyamoto 2008 [81] | 18 |  |  |
| Mo 2018 [30] | 5278 | 61 | 53.00 |
| Moon 2016 [82] | 2549 | 63.49 |  |
| Nagl 2014 [83] | 37 | 18.3 |  |
| Nazim 2014 [84] | 438 | 8.77 | 55.02 |
| Nichols 2010 [85] | 2417 | 60.2 | 48.60 |
| Noguez 2013 [86] | 4380 |  |  |
| Parkes 1999 [87] | 59 |  |  |
| Parrinello 2015 [88] | 61 | 76 | 27.00 |
| Pecoraro 1986 [89] | 11 | 62.3 | 97.56 |
| Penno 2013 [90] | 6157 | 66.4 | 53.60 |
| Phillipou 1993 [91] | 16 | 48 | 18.75 |
| Pimentel 2017 [92] | 95 | 43.6 | 42.11 |
| Prentice 2016 [93] | 50,861 | 65.64 | 98.00 |
| Ramachandran 2015 [94] | 632 | 51.7 | 45.40 |
| Raman 2016 [20] | 1195 | 14.7 | 53.00 |
| Rodriguez-Segade 2012 [95] | 2103 | 59.2 | 47.70 |
| Rohlfing 2002 [96] | 48 |  |  |
| Romero-Aroca 2021 [97] | 260 | 36 | 51.53 |
| Rosa 2019 [98] | 220 | 29.6 | 40.00 |
| Rutter 2022 [99] | 12860 |  |  |
| Sakai 2020 [100] | 301 | 68 | 65.78 |
| Sambataro 2017 [101] | 39 | 60 |  |
| Savu 2016 [102] | 14 | 62.7 | 57.14 |
| Schreur 2018 [103] | 131 | 48 | 46.00 |
| Scott 2018 [104] | 9795 |  |  |
| Scott 2019 [105] | 506 | 38 | 50.00 |
| Segar 2020 [17] | 8576 | 62.7 | 61.60 |
| Selvin 2007 [106] | 685 | 47.2 | 49.80 |
| Sheedy 2022 [107] | 1102 |  |  |
| Su 2018 [108] | 563 | 56.4 | 53.10 |
| Sugawara 2012 [18] | 619 | 54.3 | 69.00 |
| Sun 2019 [109] | 930 | 64.7 | 54.90 |
| Takahashi 2014 [110] | 34662 | 49 | 52.00 |
| Takao 2014 [111] | 754 | 54.4 | 81.80 |
| Takenouchi 2016 [112] | 162 | 62.3 | 53.60 |
| Teliti 2018 [113] | 900 | 66.96 | 57.22 |
| Thyagarajan 2016 [114] | 57 |  | 36.73 |
| Tjaden 2019 [115] | 66 | 14.2 |  |
| Trape 2000 [116] | 47 | 61.9 |  |
| Tseng 2020 [117] | 4216 | 58.6 | 49.30 |
| Tsuboi 2017 [118] | 168 | 59.4 | 61.50 |
| Tsuchiya 2020 [119] | 156 | 67 | 39.10 |
| Tynjala 2020 [120] | 718 | 46 | 46.90 |
| Ucar 2013 [121] | 29 |  | 31.03 |
| Virk 2016 [122] | 1706 | 15.9 |  |
| Waden 2009 [123] | 2107 | 36.4 | 53.20 |
| Walker 2017 [124] | 6048 | 44 |  |
| Wan 2016 [16] | 91866 | 62.58 | 44.20 |
| Watts 1989 [125] | 172 | 31.4 | 55.81 |
| Whitsel 2012 [126] | 100 |  |  |
| Wightman 2018 [127] | 5952 | 44 |  |
| Yang 2020 [128] | 1705 | 53.14 | 57.42 |
| Yang 2018 [129] | 681 | 58.9 | 60.10 |
| Yang 2015a [130] | 31841 | 60.94 |  |
| Yang 2015b [131] | 595 | 64.88 | 58.32 |
| Yu 2020 [21] | 3254 | 65 | 39.34 |
| Zhong 2018 [132] | 193 | 52.17 | 56.48 |

**S1 Table 5 Characteristics of papers (continued)**

| Paper | Setting  code | Health status code | Average number of measurements (primary group) | CV_I_ or CV_T_ recorded |
| --- | --- | --- | --- | --- |
| Agard 1997 [33] | 2 | 3 | 16 | T |
| Barton 2019 [34] |  | 2 | 3 | T |
| Borsey 1982 [35] |  | 2 | 6 | T |
| Bouchi 2012 [36] | 2 | 2 | 26 | T |
| Braga 2011 [37] | 2 | 1 | 5 | I |
| Cardoso 2018 [38] | 2 | 2 | 12 | T |
| Carlsen 2011 [39] | 2 | 1 | 10 | T |
| Ceriello 2017 [40] | 2 | 2 | 5 | T |
| Chiu 2020 [28] | 2 | 2 |  | T |
| Chiu 2017 [41] |  | 2 |  | T |
| Coskun 2018 [42] | 2 | 1 | 4 | I |
| Critchley 2019 [43] | 1 | 2 | 4 | T |
| Dergez 2014 [44] |  | 1 | 10 | U |
| Desmeules 2010 [45] | 2 | 5 | 5 | I |
| Dorajoo 2017 [46] | 2 | 2 | 11 | T |
| Ercan 2015 [47] |  | 1 | 7 | U |
| Ercan 2018 [48] | 2 | 1 | 8 | I |
| Foo 2017 [49] | 1 | 2 | 4 | T |
| Garde 2000 [50] | 2 | 1 | 12 | T |
| Godsland 1985 [51] | 2 | 1 | 11 | I |
| Gu 2018 [52] | 2 | 2 | 11.7 | T |
| Gu 2017 [53] | 2 | 2 | 11.3 | T |
| Hashimoto 2020 [54] | 2 | 2 | 5 | T |
| He 2015 [55] |  | 1 | 5 | U |
| Herath 2016 [56] |  | 2 | 2 | U |
| Hermann 2014 [57] | 2 | 3 | 5 | T |
| Hietala 2013 [58] | 2 | 3 | 2 | T |
| Hirakawa 2014 [59] | 2 | 2 | 5 | T |
| Howey 1989 [60] | 2 | 2 | 6 | I |
| Hsu 2012 [61] | 2 | 2 | 9 | T |
| Jang 2019 [62] | 2 | 1 | 5 | T |
| Jorde 2000 [63] | 2 | 3 | 10.4 | T |
| Jun 2015 [64] | 2 | 2 | 6 | T |
| Jury 1983 [65] | 2 | 2 | 18 | T |
| Kelly 2019 [66] | 1 | 1 | 2 | U |
| Kilpatrick 1998 [67] | 2 | 1 | 10 | I |
| Kilpatrick 2008 [68] | 2 | 3 |  | T |
| Kitaoka 2016 [69] |  | 2 | 12 | T |
| Lee 2020 [70] | 2 | 2 | 4 | T |
| Lenters-Westra 2015 [6] | 2 | 1 | 4 | U |
| Lenters-Westra 2014 [71] | 2 | 1 | 4 | I |
| Li 2017 [72] | 1 | 2 | 3.46 | T |
| Liang 2019 [27] | 2 | 1 | 5 | I |
| Lin 2013 [73] | 2 | 2 |  | T |
| Low 2017 [74] | 2 | 2 | 8 | T |
| Lui 2020 [29] | 2 | 2 | 7.1 | T |
| Luk 2013 [75] | 2 | 2 | 10 | T |
| Ma 2012 [25] | 2 | 2 | 12 | T |
| Matsutani 2018 [78] | 2 | 2 | 8 | T |
| Mao 2022 [77] | 1 | 3 |  | T |
| Meigs 1996 [79] | 1 | 1 | 2 | T |
| Mellergard 2020 [19] | 1 | 2 | 5 | T |
| Minami 2020 [80] | 2 | 2 |  | T |
| Miyamoto 2008 [81] | 2 | 5 | 9.1 | T |
| Mo 2018 [30] | 2 | 2 | 4 | T |
| Moon 2016 [82] |  | 2 |  | U |
| Nagl 2014 [83] |  | 3 |  | U |
| Nazim 2014 [84] | 2 | 3 | 36.8 | T |
| Nichols 2010 [85] | 2 | 2 | 12.4 | T |
| Noguez 2013 [86] |  |  |  | I |
| Parkes 1999 [87] | 2 | 4 | 2 | T |
| Parrinello 2015 [88] | 1 | 1 | 2 | U |
| Pecoraro 1986 [89] | 2 | 2 | 3 | T |
| Penno 2013 [90] | 2 | 2 | 4.52 | T |
| Phillipou 1993 [91] | 2 | 4 | 4.1 | B |
| Pimentel 2017 [92] | 2 | 5 | 3 | I |
| Prentice 2016 [93] | 1 | 2 | 4 | T |
| Ramachandran 2015 [94] | 1 | 2 | 6.3 | T |
| Raman 2016 [20] | 2 | 3 | 14 | T |
| Rodriguez-Segade 2012 [95] | 2 | 2 | 10 | T |
| Rohlfing 2002 [96] |  | 1 | 12 | I |
| Romero-Aroca 2021 [97] | 2 | 3 | 8 | T |
| Rosa 2019 [98] | 2 | 3 | 17 | U |
| Rutter 2022 [99] | 2 | 1 | 2 | T |
| Sakai 2020 [100] | 2 | 2 | 5 | T |
| Sambataro 2017 [101] |  | 2 |  | U |
| Savu 2016 [102] | 2 | 2 | 3 | T |
| Schreur 2018 [103] | 2 | 3 | 5 | T |
| Scott 2018 [104] |  | 2 | 6 | T |
| Scott 2019 [105] | 2 | 3 | 8 | T |
| Segar 2020 [17] | 2 | 2 | 8 | T |
| Selvin 2007 [106] | 1 | 1 | 2 | I |
| Sheedy 2022 [107] | 2 | 4 |  | T |
| Su 2018 [108] | 2 | 2 | 4 | T |
| Sugawara 2012 [18] | 2 | 2 | 11 | T |
| Sun 2019 [109] |  | 2 | 5 | T |
| Takahashi 2014 [110] | 2 | 1 | 5 | T |
| Takao 2014 [111] | 2 | 2 | 4 | T |
| Takenouchi 2016 [112] | 2 | 2 | 6 | T |
| Teliti 2018 [113] | 2 | 2 | 5 | T |
| Thyagarajan 2016 [114] | 1 | 1 | 2 | I |
| Tjaden 2019 [115] | 2 | 2 | 2 | T |
| Trape 2000 [116] | 2 | 2 | 4.2 | I |
| Tseng 2020 [117] | 2 | 2 | 3 | T |
| Tsuboi 2017 [118] | 2 | 2 | 12 | T |
| Tsuchiya 2020 [119] |  | 2 | 13 | T |
| Tynjala 2020 [120] | 1 | 3 | 16 | T |
| Ucar 2013 [121] |  | 1 | 4 | T |
| Virk 2016 [122] | 2 | 3 | 22 | T |
| Waden 2009 [123] | 2 | 3 | 13 | T |
| Walker 2017 [124] | 1 | 3 | 4 | T |
| Wan 2016 [16] | 1 | 2 | 6.47 | T |
| Watts 1989 [125] | 2 | 3 | 8 | T |
| Whitsel 2012 [126] | 1 | 1 | 2 | U |
| Wightman 2018 [127] | 1 | 3 | 4 | T |
| Yang 2020 [128] | 2 | 2 |  | T |
| Yang 2018 [129] |  | 2 | 16.9 | T |
| Yang 2015a [130] | 1 | 2 | 3 | T |
| Yang 2015b [131] | 2 | 2 | 4 | T |
| Yu 2020 [21] | 1 | 1 |  | T |
| Zhong 2018 [132] | 1 | 4 | 12.82 | T |

Table 3 (cont.) characteristics of papers included

Setting code: 1=primary or community;2=tertiary/secondary/laboratory

Health status code: 1=healthy 2= type 2 diabetes 3= type 1 diabetes 4= both type 1 diabetes and type 2 diabetes 5=other disease

CV_I_ or CV_T_ recorded code: I= CV_I_; T=CV_T_; U= Uncertain

# Appendix 5

**S1 table 6: Risk of bias scores**

| Paper | Risk of Bias |
| --- | --- |
| Agard 1997 [33] | 2 |
| Barton 2019 [34] | 3 |
| Borsey 1982 [35] | 1 |
| Bouchi 2012 [36] | 1 |
| Braga 2011 [37] | 1 |
| Cardoso 2018 [38] | 1 |
| Carlsen 2011 [39] | 1 |
| Ceriello 2017 [40] | 3 |
| Chiu 2020 [28] | 2 |
| Chiu 2017 [41] | 2 |
| Coskun 2018 [42] | 1 |
| Critchley 2019 [43] | 2 |
| Dergez 2014 [44] | 2 |
| Desmeules 2010 [45] | 1 |
| Dorajoo 2017 [46] | 1 |
| Ercan 2015 [47] | 3 |
| Ercan 2018 [48] | 1 |
| Foo 2017 [49] | 2 |
| Garde 2000 [50] | 1 |
| Godsland 1985 [51] | 1 |
| Gu 2018 [52] | 3 |
| Gu 2017 [53] | 2 |
| Hashimoto 2020 [54] | 2 |
| He 2015 [55] | 2 |
| Herath 2016 [56] | 3 |
| Hermann 2014 [57] | 2 |
| Hietala 2013 [58] | 2 |
| Hirakawa 2014 [59] | 1 |
| Howey 1989 [60] | 2 |
| Hsu 2012 [61] | 2 |
| Jang 2019 [62] | 1 |
| Jorde 2000 [63] | 1 |
| Jun 2015 [64] | 1 |
| Jury 1983 [65] | 3 |
| Kelly 2019 [66] | 2 |
| Kilpatrick 1998 [67] | 1 |
| Kilpatrick 2008 [68] | 2 |
| Kitaoka 2016 [69] | 2 |
| Lee 2020 [70] | 2 |
| Lenters-Westra 2015 [6] | 2 |
| Lenters-Westra 2014 [71] | 3 |
| Li 2017 [72] | 3 |
| Liang 2019 [27] | 1 |
| Lin 2013 [73] | 2 |
| Low 2017 [74] | 2 |
| Lui 2020 [29] | 2 |
| Luk 2013 [75] | 3 |
| Ma 2012 [25] | 2 |
| Mao 2022 [77] | 2 |
| Matsutani 2018 [78] | 3 |
| Meigs 1996 [79] | 2 |
| Mellergard 2020 [19] | 3 |
| Minami 2020 [80] | 2 |
| Miyamoto 2008 [81] | 3 |
| Mo 2018 [30] | 3 |
| Moon 2016 [82] | 3 |
| Nagl 2014 [83] | 3 |
| Nazim 2014 [84] | 2 |
| Nichols 2010 [85] | 2 |
| Noguez 2013 [86] | 3 |
| Parkes 1999 [87] | 3 |
| Parrinello 2015 [88] | 3 |
| Pecoraro 1986 [89] | 2 |
| Penno 2013 [90] | 2 |
| Phillipou 1993 [91] | 3 |
| Pimentel 2017 [92] | 2 |
| Prentice 2016 [93] | 3 |
| Ramachandran 2015 [94] | 3 |
| Raman 2016 [20] | 2 |
| Rodriguez-Segade 2012 [95] | 2 |
| Rohlfing 2002 [96] | 3 |
| Romero-Aroca 2021 [97] | 2 |
| Rosa 2019 [98] | 3 |
| Rutter 2022 [99] | 1 |
| Sakai 2020 [100] | 2 |
| Sambataro 2017 [101] | 3 |
| Savu 2016 [102] | 3 |
| Schreur 2018 [103] | 2 |
| Scott 2018 [104] | 2 |
| Scott 2019 [105] | 2 |
| Segar 2020 [17] | 2 |
| Selvin 2007 [106] | 1 |
| Sheedy 2022 [107] | 2 |
| Su 2018 [108] | 2 |
| Sugawara 2012 [18] | 2 |
| Sun 2019 [109] | 2 |
| Takahashi 2014 [110] | 3 |
| Takao 2014 [111] | 2 |
| Takenouchi 2016 [112] | 3 |
| Teliti 2018 [113] | 3 |
| Thyagarajan 2016 [114] | 2 |
| Tjaden 2019 [115] | 3 |
| Trape 2000 [116] | 1 |
| Tseng 2020 [117] | 3 |
| Tsuboi 2017 [118] | 2 |
| Tsuchiya 2020 [119] | 3 |
| Tynjala 2020 [120] | 2 |
| Ucar 2013 [121] | 2 |
| Virk 2016 [122] | 2 |
| Waden 2009 [123] | 2 |
| Walker 2017 [124] | 3 |
| Wan 2016 [16] | 2 |
| Watts 1989 [125] | 3 |
| Whitsel 2012 [126] | 3 |
| Wightman 2018 [127] | 3 |
| Yang 2020 [128] | 3 |
| Yang 2018 [129] | 2 |
| Yang 2015a [130] | 2 |
| Yang 2015b [131] | 2 |
| Yu 2020 [21] | 1 |
| Zhong 2018 [132] | 3 |

Table 4: Risk of bias estimates for each included paper, with questions 4 and 5 excluded. Highest score presented. 1=very good; 2=adequate; 3=doubtful; 4=inadequate.

# Appendix 6

## Statistical calculations for ICC meta-analysis

Z = 0.5 * ln((1 + ICC) / (1 - ICC)). Standard error was calculated using the formula SE = sqrt(1/(N - 3) Z scores were then back-transformed

## Stata code

### For ICC:

sort Totalsubjectsinprimaryanalys

gen z = 0.5 * ln((1 + ICCprimarygroup ) / (1 - ICCprimarygroup ))

gen sez = sqrt(1/( Totalsubjectsinprimaryanalys - 3))

meta set z sez, random(dl) studylabel( Shorttitle ) studysize( Totalsubjectsinprimaryanalys )

meta forestplot

gen iccbk = (exp(2*z) - 1)/(exp(2*z) + 1)

gen cilicc = (exp(2* _meta_cil ) - 1)/(exp(2* _meta_cil ) + 1)

gen ciuicc = (exp(2* _meta_ciu ) - 1)/(exp(2* _meta_ciu ) + 1)

metan iccbk cilicc ciuicc if Unitusedforprimaryanalysis ==1 , random by( Healthstatusshortname ) label( namevar=Shorttitle) rcols ( Totalsubjectsinprimaryanalys ) xlabel (0, 0.2, 0.4, 0.6, 0.8, 1.0) xtitle ("ICC")

### For CV

sort Totalsubjectsinprimaryanalys

label variable PrimarymeasureofCVIaverage "CVI"

rename PrimarymeasureofCVIaverage CVI

label variable Healthstatusshortname "Health status"

label variable Shorttitle "Study title"

label variable Averagenumberofmeasurements "Number of measurements"

label variable Totalsubjectsinprimaryanalys "Number of subjects"

generate cil = CVI - CVI * 0.0001

generate ciu = CVI + CVI * 0.0001

metan CVI cilc ciu if Unitusedforprimaryanalysis ==1 , randomi by( Healthstatusshortname ) label( namevar=Shorttitle) rcols ( CVI Averagenumberofmeasurement Totalsubjectsinprimaryanalys ) nosubgroup nostats xlabel (0, 0.05, 0.1, 0.15, 0.2) nooverall nowt nobox xtitle (CV)

metan CVI cilc ciuc if ( Unitusedforprimaryanalysis ==1 & Healthstatuscode ==1 ), randomi label( namevar=Shorttitle) rcols ( CVI Averagenumberofmeasurement Totalsubjectsinprimaryanalys ) nosubgroup nostats xlabel (0, 0.02, 0.04, 0.06, 0.08, 0.10) nooverall nowt nobox xtitle (CV)

metan CVI cil ciu if Healthstatuscode ==2 , randomi by( Unitusedforprimaryanalysis ) label( namevar=Shorttitle) rcols ( CVI Averagenumberofmeasurement Totalsubjectsinprimaryanalys ) nosubgroup nostats xlabel (0, 0.02, 0.04, 0.06, 0.08) nooverall nowt nobox xtitle (CV)

# Appendix 7

Forest plots of subgroup and sensitivity analyses


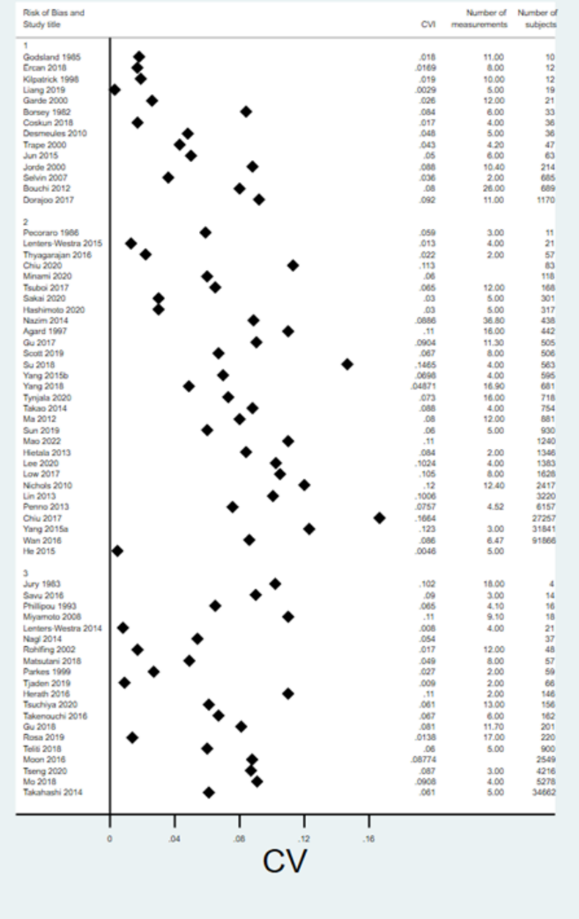


**S1Fig 1 Forest plot of CV for studies reporting units of %, split by risk of bias**


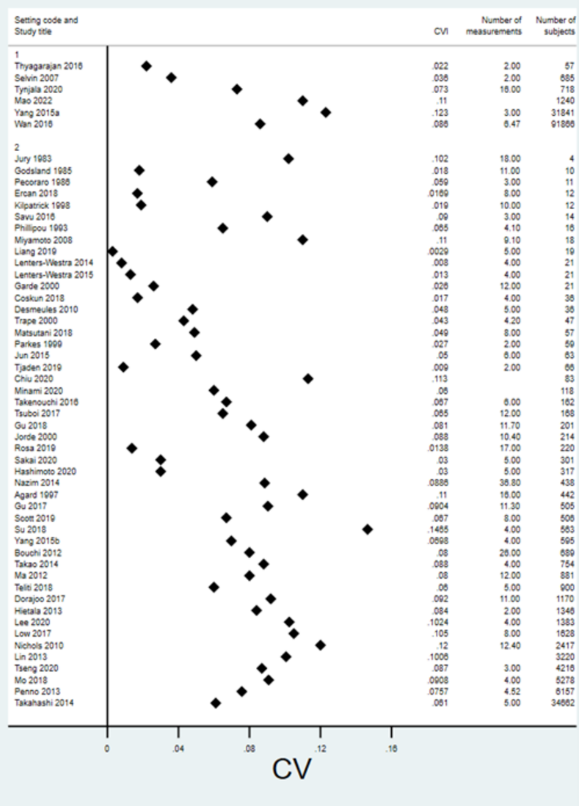


**S1Fig2 Forest plot of CV for studies reporting units of %, split by setting**. 1=primary care or community, 2=secondary or tertiary care


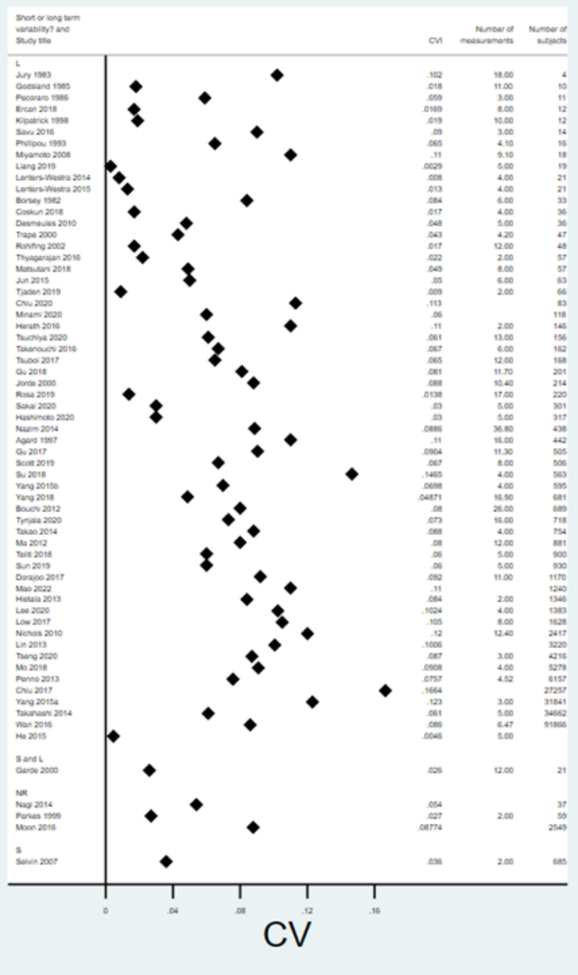


**S1Fig3 Forest plot of CV for studies reporting units of %, split by short (S) or long (L) term measurements**


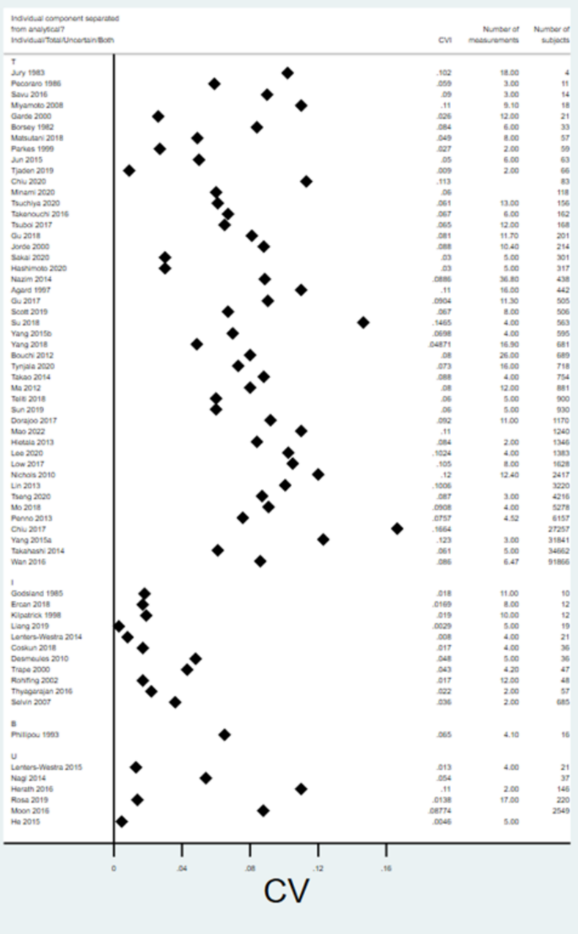


**S1Fig 4 Forest plot of CV for studies reporting units of %, split by whether CV_I_ (I) or CV_T_ (T) was reported or whether this was uncertain (U)**

# Appendix 8

Full reference list

1. Barr RG, David MN, James BM, Daniel ES. Tests of Glycemia for the Diagnosis of Type 2 Diabetes Mellitus. Ann Intern Med. 2002;137(4):263.

2. American Diabetes Association. 2. Classification and Diagnosis of Diabetes: Standards of Medical Care in Diabetes—2021. Diabetes Care. 2020;44(Supplement_1):S15-S33.

3. Anonymous. Type 2 diabetes in adults: management, NICE guideline [NG28] 2022 [Available from: https://www.nice.org.uk/guidance/ng28/chapter/Recommendations.

4. Lenters-Westra E, English E. Evaluating new HbA1c methods for adoption by the IFCC and NGSP reference networks using international quality targets. Clin Chem Lab Med. 2017;55(9):1426-34.

5. Dhatt GS, Agarwal MM, Bishawi B. HbA1c: A comparison of NGSP with IFCC transformed values. Clinica Chimica Acta. 2005;358(1):81-6.

6. Lenters-Westra E, Roraas T, Schindhelm RK, Slingerland RJ, Sandberg S. Biological variation of Hemoglobin A1c: Consequences for diagnosing diabetes mellitus. Nederlands Tijdschrift voor Klinische Chemie en Laboratoriumgeneeskunde. 2015;40 (2):104.

7. Little RR, Rohlfing CL. The long and winding road to optimal HbA1c measurement. Clin Chim Acta. 2013;418:63-71.

8. Raphael A, Friger M, Biderman A. Seasonal variations in HbA1c among type 2 diabetes patients on a semi-arid climate between the years 2005-2015. Prim Care Diabetes. 2021;15(3):502-6.

9. Fraser Callum G. Biological Variation: From Principles to Practice. USA: AACC Press; 2001.

10. Gonzalez-Lao E, Corte Z, Simon M, Ricos C, Coskun A, Braga F, et al. Systematic review of the biological variation data for diabetes related analytes. Clinica Chimica Acta. 2019;488:61-7.

11. Anonymous. European Federation of Clinical Chemistry and Laboratory Medicine (EFLM) Biological Variation Database 2022 [Available from: https://biologicalvariation.eu/about.

12. Braga F, Dolci A, Mosca A, Panteghini M, Sacco L, editors. Variabilità biologica delŒ emoglobina glicata: un analisi sistematica della letteratura2009.

13. Byron C. Wallace KS, Carla E. Brodley, Joseph Lau and Thomas A. Trikalinos, editor Deploying an interactive machine learning system in an evidence-based practice center: abstrackr. ACM International Health Informatics Symposium (IHI); 2012.

14. Mokkink LB, Boers M, van der Vleuten CPM, Bouter LM, Alonso J, Patrick DL, et al. COSMIN Risk of Bias tool to assess the quality of studies on reliability or measurement error of outcome measurement instruments: a Delphi study. BMC Med Res Methodol. 2020;20(1):293.

15. Field AP. Is the meta-analysis of correlation coefficients accurate when population correlations vary? Psychol Methods. 2005;10(4):444-67.

16. Wan EYF. Association of variability in hemoglobin A1c with cardiovascular diseases and mortality in Chinese patients with type 2 diabetes mellitus — A retrospective population-based cohort study. Journal of Diabetes & its Complications. 2016;30(7):1240-8.

17. Segar MW, Patel KV, Vaduganathan M, Caughey MC, Butler J, Fonarow GC, et al. Association of Long-term Change and Variability in Glycemia With Risk of Incident Heart Failure Among Patients With Type 2 Diabetes: A Secondary Analysis of the ACCORD Trial. Diabetes care. 2020;15.

18. Sugawara A, Kawai K, Motohashi S, Saito K, Kodama S, Yachi Y, et al. HbA1c variability and the development of microalbuminuria in type 2 diabetes: Tsukuba Kawai Diabetes Registry 2. Diabetologia. 2012;55(8):2128-31.

19. Mellergard E, Johnsson P, Eek F. Sociodemographic factors associated with HbA1c variability in type 2 diabetes: a prospective exploratory cohort study. BMC Endocrine Disorders. 2020;20(1).

20. Raman S, Dai H, DeLurgio SA, Williams DD, Lind M, Patton SR, et al. High hemoglobin A1c variability is associated with early risk of microalbuminuria in children with T1D. Pediatr Diabetes. 2016;17(6):398-406.

21. Yu ZB, Zhu Y, Li D, Wu MY, Tang ML, Wang JB, et al. Association between visit-to-visit variability of HbA<sub>1c</sub> and cognitive decline: a pooled analysis of two prospective population-based cohorts. Diabetologia. 2020;63(1):85-94.

22. Carlsen S, Petersen PH, Skeie S, Skadberg O, Sandberg S. Within-subject biological variation of glucose and HbA(1c) in healthy persons and in type 1 diabetes patients. Clinical Chemistry & Laboratory Medicine. 2011;49(9):1501-7.

23. Maguire A, Mitchell BD, Ruzafa JC. Antihyperglycaemic treatment patterns, observed glycaemic control and determinants of treatment change among patients with type 2 diabetes in the United Kingdom primary care: a retrospective cohort study. BMC Endocr Disord. 2014;14:73.

24. Hirst JA, Farmer AJ, Ali R, Roberts NW, Stevens RJ. Quantifying the effect of metformin treatment and dose on glycemic control. Diabetes Care. 2012;35(2):446-54.

25. Ma WY, Li HY, Pei D, Hsia TL, Lu KC, Tsai LY, et al. Variability in hemoglobin A1c predicts all-cause mortality in patients with type 2 diabetes. Journal of Diabetes & its Complications. 2012;26(4):296-300.

26. Caturano A, Galiero R, Pafundi PC, Cesaro A, Vetrano E, Palmiero G, et al. Does a strict glycemic control during acute coronary syndrome play a cardioprotective effect? Pathophysiology and clinical evidence. Diabetes Res Clin Pract. 2021;178:108959.

27. Liang L, He H, Zeng Y, Zhang M, Wang X, Li X, et al. Evaluation of biological variation of glycated hemoglobin and glycated albumin in healthy Chinese subjects. Journal of Clinical Laboratory Analysis. 2019;33(3):e22715.

28. Chiu WC, Lai YR, Cheng BC, Huang CC, Chen JF, Lu CH. HbA1C Variability Is Strongly Associated with Development of Macroalbuminuria in Normal or Microalbuminuria in Patients with Type 2 Diabetes Mellitus: A Six-Year Follow-Up Study. BioMed Research International. 2020;2020 (no pagination).

29. Lui DTW, Lee CH, Chan YH, Chow WS, Fong CHY, Siu DCW, et al. HbA1c variability, in addition to mean HbA1c, predicts incident hip fractures in Chinese people with type 2 diabetes. Osteoporosis International. 2020.

30. Mo Y, Zhou J, Ma X, Zhu W, Zhang L, Li J, et al. Haemoglobin A1c variability as an independent correlate of atherosclerosis and cardiovascular disease in Chinese type 2 diabetes. Diabetes & Vascular Disease Research. 2018;15(5):402-8.

31. Cavagnolli G, Pimentel AL, Freitas PAC, Gross JL, Camargo JL. Effect of ethnicity on HbA1c levels in individuals without diabetes: Systematic review and meta-analysis. PLoS One. 2017;12(2):e0171315-e.

32. Koo TK, Li MY. A Guideline of Selecting and Reporting Intraclass Correlation Coefficients for Reliability Research. J Chiropr Med. 2016;15(2):155-63.

33. Agardh CD, Agardh E, Torffvit O. The association between retinopathy, nephropathy, cardiovascular disease and long-term metabolic control in type 1 diabetes mellitus: a 5 year follow-up study of 442 adult patients in routine care. Diabetes Research & Clinical Practice. 1997;35(2-3):113-21.

34. Barton K, Donovan L, Giroux I, Miller D, Mottola M, McManus R. Glycated hemoglobin measurements at three, 12 and 24 months postpartum after gestational diabetes. Clinical and investigative medicine. 2019;Medecine clinique et experimentale. 42(4):E37-E41.

35. Borsey DQ, Fraser DM, Gray RS. Glycosylated hemoglobin and its temporal relationship to plasma glucose in non-insulin dependent (type 2) diabetes mellitus. Metabolism: Clinical and Experimental. 1982;31(4):362-5.

36. Bouchi R, Babazono T, Mugishima M, Yoshida N, Nyumura I, Toya K, et al. Fluctuations in HbA1c are associated with a higher incidence of cardiovascular disease in Japanese patients with type 2 diabetes. Journal of Diabetes Investigation. 2012;3(2):148-55.

37. Braga F, Dolci A, Montagnana M, Pagani F, Paleari R, Guidi GC, et al. Revaluation of biological variation of glycated hemoglobin (HbA1c) using an accurately designed protocol and an assay traceable to the IFCC reference system. Clinica Chimica Acta. 2011;412(15-16):1412-6.

38. Cardoso CRL, Leite NC, Moram CBM, Salles GF. Long-term visit-to-visit glycemic variability as predictor of micro- and macrovascular complications in patients with type 2 diabetes: The Rio de Janeiro Type 2 Diabetes Cohort Study. Cardiovascular Diabetology. 2018;17(1).

39. Carlsen S, Petersen PH, Skeie S, Oyvind S, Sandberg S. Within-subject biological variation of glucose and HbA1c in healthy persons and in type 1 diabetes patients. Clinical Chemistry and Laboratory Medicine. 2011;49(9):1501-7.

40. Ceriello A, De Cosmo S, Rossi MC, Lucisano G, Genovese S, Pontremoli R, et al. Variability in HbA1c, blood pressure, lipid parameters and serum uric acid, and risk of development of chronic kidney disease in type 2 diabetes. Diabetes Obes Metab. 2017;19(11):1570-8.

41. Chiu HT, Li TC, Li CI, Liu CS, Lin WY, Lin CC. Visit-to-visit glycemic variability is a strong predictor of chronic obstructive pulmonary disease in patients with type 2 diabetes mellitus: Competing risk analysis using a national cohort from the Taiwan diabetes study. PLoS ONE. 2017;12(5).

42. Coskun A, Carobene A, Kilercik M, Serteser M, Sandberg S, Aarsand AK, et al. Within-subject and between-subject biological variation estimates of 21 hematological parameters in 30 healthy subjects. Clinical Chemistry and Laboratory Medicine. 2018;56(8):1309-18.

43. Critchley JA, Carey IM, Harris T, DeWilde S, Cook DG. Variability in glycated hemoglobin and risk of poor outcomes among people with type2diabetesinalargeprimary care cohort study. Diabetes Care. 2019;42(12):2237-46.

44. Dergez E, Lanyi E. Calculation of a reference change value with application of locally estimated biological variation of Hemoglobin A1c. Clinical Chemistry and Laboratory Medicine. 2014;52 (9):eA97.

45. Desmeules P, Cousineau J, Allard P. Biological variation of glycated haemoglobin in a paediatric population and its application to calculation of significant change between results. Ann Clin Biochem. 2010;47(Pt 1):35-8.

46. Dorajoo SR, Ng JSL, Goh JHF, Lim SC, Yap CW, Chan A, et al. HbA1c variability in type 2 diabetes is associated with the occurrence of new-onset albuminuria within three years. Diabetes Research and Clinical Practice. 2017;128:32-9.

47. Ercan M, Akbulut D, Oguz E, Bal C, Cicek EA, Yilmaz FM. Estimation of biological variation components of glycated hemoglobin HbA1c on Tosoh G7 HPLC. Turkish Journal of Biochemistry. 2015;40 (Supplement 1):157.

48. Ercan M, Avci E, Akbulut ED, Oguz EF, Topcuoglu C, Yucel C, et al. Biological variation components of glycated hemoglobin HbA1C on tosoh G7 HPLC: Biological variation components of HbA1C. Journal of Clinical and Analytical Medicine. 2018;9(6):469-73.

49. Foo V, Quah J, Cheung G, Tan NC, Ma Zar KL, Chan CM, et al. HbA1c, systolic blood pressure variability and diabetic retinopathy in Asian type 2 diabetics. J Diabetes. 2017;9(2):200-7.

50. Garde AH, Hansen AM, Skovgaard LT, Christensen JM. Seasonal and biological variation of blood concentrations of total cholesterol, dehydroepiandrosterone sulfate, hemoglobin A(1c), IgA, prolactin, and free testosterone in healthy women. Clinical Chemistry. 2000;46(4):551-9.

51. Godsland IF. Intra-individual variation: significant changes in parameters of lipid and carbohydrate metabolism in the individual and intra-individual variation in different test populations. Annals of Clinical Biochemistry. 1985;22(Pt 6):618-24.

52. Gu J, Fan YQ, Zhang JF, Wang CQ. Association of hemoglobin A1c variability and the incidence of heart failure with preserved ejection fraction in patients with type 2 diabetes mellitus and arterial hypertension. Hellenic Journal of Cardiology. 2018;59(2):91-7.

53. Gu J, Fan YQ, Zhang JF, Wang CQ. Impact of long-term glycemic variability on development of atrial fibrillation in type 2 diabetic patients. Anatolian Journal of Cardiology. 2017;18(6):410-6.

54. Hashimoto Y, Kaji A, Sakai R, Osaka T, Ushigome E, Hamaguchi M, et al. Skipping breakfast is associated with glycemic variability in patients with type 2 diabetes. Nutrition. 2020;71 (no pagination).

55. He H, Peng S, Liang L, Zhou J, An Z, Huang H. Biological variation and reference change value of HbA1c and glycated albumin in healthy individuals during short-term follow-up. Clinical Chemistry and Laboratory Medicine. 2015;53:S603.

56. Herath M, Weerarathna TP. Lack of association between different metrics of glycemic control with progression of nephropathy in type 2 diabetes: A preliminary observation from a Sri Lankan cohort. Diabetes. 2016;65 (Supplement 1):A550.

57. Hermann JM, Hammes H-P, Rami-Merhar B, Rosenbauer J, Schütt M, Siegel E, et al. HbA1c Variability as an Independent Risk Factor for Diabetic Retinopathy in Type 1 Diabetes: A German/Austrian Multicenter Analysis on 35,891 Patients. PLOS ONE. 2014;9(3):e91137.

58. Hietala K, Wadén J, Forsblom C, Harjutsalo V, Kytö J, Summanen P, et al. HbA1c variability is associated with an increased risk of retinopathy requiring laser treatment in type 1 diabetes. Diabetologia. 2013;56(4):737-45.

59. Hirakawa Y, Arima H, Zoungas S, Ninomiya T, Cooper M, Hamet P, et al. Impact of visit-to-visit glycemic variability on the risks of macrovascular and microvascular events and all-cause mortality in type 2 diabetes: The ADVANCE trial. Diabetes Care. 2014;37(8):2359-65.

60. Howey JE, Bennet WM, Browning MC, Jung RT, Fraser CG. Clinical utility of assays of glycosylated haemoglobin and serum fructosamine compared: use of data on biological variation. Diabetic Medicine. 1989;6(9):793-6.

61. Hsu CC, Chang HY, Huang MC, Hwang SJ, Yang YC, Lee YS, et al. HbA1c variability is associated with microalbuminuria development in type 2 diabetes: a 7-year prospective cohort study. Diabetologia. 2012;55(12):3163-72.

62. Jang JY, Moon S, Cho S, Cho KH, Oh CM. Visit-to-visit HbA1c and glucose variability and the risks of macrovascular and microvascular events in the general population. Scientific reports. 2019;9(1):1374.

63. Jorde R, Sundsfjord J. Intra-individual variability and longitudinal changes in glycaemic control in patients with Type 1 diabetes mellitus. Diabetic Medicine. 2000;17(6):451-6.

64. Jun JE, Jin SM, Baek J, Oh S, Hur KY, Lee MS, et al. The association between glycemic variability and diabetic cardiovascular autonomic neuropathy in patients with type 2 diabetes. Cardiovascular Diabetology. 2015;14(1).

65. Jury DR, Baker JR, Dunn PJ. Clinical importance of the reversible fraction of haemoglobin A1c in Type 2 (non-insulin-dependent) diabetes. Diabetologia. 1983;25(4):313-5.

66. Kelly K, Mezuk B. Depression, Inflammation, and Metabolic Risk: A Genetically-Informed Exploratory Study. European Neuropsychopharmacology. 2019;29 (Supplement 3):S976-S7.

67. Kilpatrick ES, Maylor PW, Keevil BG. Biological variation of glycated hemoglobin. Implications for diabetes screening and monitoring. Diabetes Care. 1998;21(2):261-4.

68. Kilpatrick ES, Rigby AS, Atkin SL. A1C variability and the risk of microvascular complications in type 1 diabetes: data from the Diabetes Control and Complications Trial. Diabetes Care. 2008;31(11):2198-202.

69. Kitaoka K, Takenouchi A, Tsuboi A, Fukuo K, Kazumi T. Association of Postbreakfast Triglyceride and Visit-to-Visit Annual Variation of Fasting Plasma Glucose with Progression of Diabetic Nephropathy in Patients with Type 2 Diabetes. Journal of Diabetes Research. 2016;2016:4351376.

70. Lee CL, Chen CH, Wu MJ, Tsai SF. The variability of glycated hemoglobin is associated with renal function decline in patients with type 2 diabetes. Therapeutic Advances in Chronic Disease. 2020;11(no pagination).

71. Lenters-Westra E, Roraas T, Schindhelm RK, Slingerland RJ, Sandberg S. Biological variation of hemoglobin A1c: consequences for diagnosing diabetes mellitus. Clinical Chemistry. 2014;60(12):1570-2.

72. Li TC, Yang CP, Tseng ST, Li CI, Liu CS, Lin WY, et al. Visit-to-visit variations in fasting plasma glucose and HbA1c associated with an increased risk of Alzheimer disease: Taiwan diabetes study. Diabetes Care. 2017;40(9):1210-7.

73. Lin CC, Chen CC, Chen FN, Li CI, Liu CS, Lin WY, et al. Risks of diabetic nephropathy with variation in hemoglobin A1c and fasting plasma glucose. American Journal of Medicine. 2013;126(11):1017.e1-.e10.

74. Low S, Lim SC, Yeoh LY, Liu YL, Liu JJ, Fun S, et al. Effect of long-term glycemic variability on estimated glomerular filtration rate decline among patients with type 2 diabetes mellitus: Insights from the Diabetic Nephropathy Cohort in Singapore. Journal of Diabetes. 2017;9(10):908-19.

75. Luk AOY, Ma RCW, Lau ESH, Yang X, Lau WWY, Yu LWL, et al. Risk association of HbA1c variability with chronic kidney disease and cardiovascular disease in type 2 diabetes: prospective analysis of the Hong Kong Diabetes Registry. Diabetes Metab Res Rev. 2013;29(5):384-90.

76. Ma CJ, Sun LC, Chen FM, Lu CY, Shih YL, Tsai HL, et al. A double-blind randomized study comparing the efficacy and safety of a composite vs a conventional intravenous fat emulsion in postsurgical gastrointestinal tumor patients. Nutrition in Clinical Practice. 2012;27(3):410-5.

77. Mao Y, Zhong W. HbA1c Variability as an Independent Risk Factor for Microvascular Complications in Type 1 Diabetes. Journal of Diabetes Science and Technology. 2022.

78. Matsutani D, Sakamoto M, Iuchi H, Minato S, Suzuki H, Kayama Y, et al. Glycemic variability in continuous glucose monitoring is inversely associated with baroreflex sensitivity in type 2 diabetes: A preliminary report. Cardiovascular Diabetology. 2018;17(1).

79. Meigs JB, Nathan DM, Cupples LA, Wilson PWF, Singer DE. Tracking of glycated hemoglobin in the original cohort of the Framingham Heart Study. Journal of Clinical Epidemiology. 1996;49(4):411-7.

80. Minami T, Ito Y, Yamada M, Furuta R, Minagawa F, Kamata K, et al. The effect of long-term past glycemic control on executive function among patients with type 2 diabetes mellitus. Diabetology International. 2020;11(2):114-20.

81. Miyamoto H, Suzuki H, Yokoyama Y, Akizuki S, Hirai N, Ohnishi A. Relationship between glycated albumin (GA) and glycated hemoglobin (A1c) in 255 patients with liver diseases using cross-sectional laboratory data. [Japanese]. Rinsho byori. 2008;The Japanese journal of clinical pathology. 56(9):761-6.

82. Moon D, Chung WS, Yoo KD. Variability in haemoglobin A1c predicts clinical outcomes in patients with asymptomatic type 2 diabetes. EuroIntervention. 2016;France.:315.

83. Nagl K, Gericke G, Duffles E, Fritsch M, Hortenhuber T, Rami-Merhar B, et al. Endothelial progenitor cells show relation to asymmetric dimethylarginine (ADMA) levels and HbA1c variability in young patients with type 1 without complications. Diabetologia. 2014;57(1):S526.

84. Nazim J, Fendler W, Starzyk J. Metabolic control and its variability are major risk factors for microalbuminuria in children with type 1 diabetes. Endokrynologia Polska. 2014;65(2):83-9.

85. Nichols GA, Gandra SR, Chiou CF, Anthony MS, Alexander-Bridges M, Brown JB. Successes and challenges of insulin therapy for type 2 diabetes in a managed-care setting. Current Medical Research & Opinion. 2010;26(1):9-15.

86. Molinaro RJ, Noguez JH. Hb A1c Testing at Proper Frequencies Does not Depict Appropriate Changes in Treatment. American Journal of Clinical Pathology. 2013;140(suppl_1):A121-A.

87. Parkes J, Ray R, Kerestan S, Davis H, Ginsberg B. Prospective evaluation of accuracy, precision, and reproducibility of an at-home hemoglobin A1c sampling kit. Diabetes technology & therapeutics. 1999;1(4):411-9.

88. Parrinello CM, Lutsey P, Couper D, Eckfeldt JH, Coresh J, Selvin E. Short-term total variability in nontraditional biomarkers of hyperglycemia in older adults. Diabetes. 2015;64:A433-A4.

89. Pecoraro RE, Koepsell TD, Chen MS. Comparative clinical reliability of fasting plasma glucose and glycosylated hemoglobin in non-insulin-dependent diabetes mellitus. Diabetes Care. 1986;9(4):370-5.

90. Penno G, Solini A, Zoppini G, Orsi E, Fondelli C, Zerbini G, et al. Hemoglobin A1c variability as an independent correlate of cardiovascular disease in patients with type 2 diabetes: a cross-sectional analysis of the Renal Insufficiency and Cardiovascular Events (RIACE) Italian Multicenter Study. Cardiovascular Diabetology. 2013;12(1):98.

91. Phillipou G, Phillips PJ. Intraindividual variation of glycohemoglobin: implications for interpretation and analytical goals. Clinical Chemistry. 1993;39(11 Pt 1):2305-8.

92. Pimentel AL, Camargo JL. Variability of glycated hemoglobin levels in the first year post renal transplantation in patients without diabetes. Clinical Biochemistry. 2017;50(18):997-1001.

93. Prentice JC, Pizer SD, Conlin PR. Identifying the independent effect of HbA<inf>1c</inf> variability on adverse health outcomes in patients with Type 2 diabetes. Diabetic Medicine. 2016;33(12):1640-8.

94. Ramachandran A, Winter M, Mann DM. Association of visit-to-visit variability of hemoglobin A1c and medication adherence. Journal of Managed Care & Specialty Pharmacy. 2015;21(3):229-37.

95. Rodriguez-Segade S, Rodriguez J, Garcia Lopez JM, Casanueva FF, Camina F. Estimation of the glycation gap in diabetic patients with stable glycemic control. Diabetes Care. 2012;35(12):2447-50.

96. Rohlfing C, Wiedmeyer HM, Little R, Grotz VL, Tennill A, England J, et al. Biological variation of glycohemoglobin. Clinical Chemistry. 2002;48(7):1116-8.

97. Romero-Aroca P, Navarro-Gil R, Feliu A, Valls A, Moreno A, Baget-Bernaldiz M. The Effect of HbA1c Variability as a Risk Measure for Microangiopathy in Type 1 Diabetes Mellitus. Diagnostics. 2021;11(7):24.

98. Rosa LCGFD, Zajdenverg L, Souto DL, Dantas JR, Pinto MVR, Salles GFDCMD, et al. HbA1c variability and long-term glycemic control are linked to diabetic retinopathy and glomerular filtration rate in patients with type 1 diabetes and multiethnic background. Journal of Diabetes and its Complications. 2019;33(9):610-5.

99. Rutter CE, Millard LAC, Borges MC, Lawlor DA. Exploring regression dilution bias using repeat measurements of 2858 variables in up to 49 000 UK Biobank participants. medRxiv. 2022;15.

100. Sakai R, Hashimoto Y, Hamaguchi M, Ushigome E, Okamura T, Majima S, et al. Living alone is associated with visit-to-visit hba1c variability in men but not in women in people with type 2 diabetes: Kamogawa-dm cohort study. Endocrine Journal. 2020;67(4):419-26.

101. Sambataro M, Tura A, Sambado L, Trevisiol E, Seganfreddo E, Durante E, et al. Possible clinical prognostic meaning of glucose variability indexes in type 2 diabetes (T2D) with lower-limb neuropathic and neuroischemic lesions-Italian leukemia association treviso section project. Diabetes. 2017;66 (Supplement 1):A590-A1.

102. Savu O, Elian V, Steriade O, Teodoru I, Mihut S, Tacu C, et al. The impact of basal insulin analogues on glucose variability in patients with type 2 diabetes undergoing renal replacement therapy for end-stage renal disease. International Urology & Nephrology. 2016;48(2):265-70.

103. Schreur V, van Asten F, Ng H, Weeda J, Groenewoud JMM, Tack CJ, et al. Risk factors for development and progression of diabetic retinopathy in Dutch patients with type 1 diabetes mellitus. Acta Opthalmologica. 2018;96(5):459-64.

104. Scott ES, Januszewski AS, O'Connell R, Colagiuri S, Fulcher GR, Keech A, et al. HbA1c coefficient of variation is an independent risk factor for chronic complications in type 2 diabetes in the FIELD study. Diabetologia. 2018;61 (Supplement 1):S519.

105. Scott ES, McGrath RT, Januszewski AS, Calandro D, Hardikar AA, O'Neal DN, et al. HbA1c variability in adults with type 1 diabetes on continuous subcutaneous insulin infusion (CSII) therapy compared to multiple daily injection (MDI) treatment. BMJ Open. 2019;9(12).

106. Selvin E, Crainiceanu CM, Brancati FL, Coresh J. Short-term variability in measures of glycemia and implications for the classification of diabetes. Archives of Internal Medicine. 2007;167(14):1545-51.

107. Sheedy C, Kyaw Tun T, McDermott JH, Sreenan S. Visit-to-visit HbA1c variability as a risk factor for retinopathy and microalbuminuria in people with diabetes. Irish Journal of Medical Science. 2022;191(Supplement 4):S120.

108. Su JB, Zhao LH, Zhang XL, Cai HL, Huang HY, Xu F, et al. HbA1c variability and diabetic peripheral neuropathy in type 2 diabetic patients. Cardiovascular Diabetology. 2018;17(1).

109. Sun B, He F, Gao Y, Zhou J, Sun L, Liu R, et al. Prognostic impact of visit-to-visit glycemic variability on the risks of major adverse cardiovascular outcomes and hypoglycemia in patients with different glycemic control and type 2 diabetes. Endocrine. 2019.

110. Takahashi O, Ohde S. The optimal interval of diabetes mellitus screening in healthy adults: A random effects model. Value Health. 2014;17 (3):A242.

111. Takao T, Matsuyama Y, Yanagisawa H, Kikuchi M, Kawazu S. Association between HbA1c variability and mortality in patients with type 2 diabetes. Journal of Diabetes and its Complications. 2014;28(4):494-9.

112. Takenouchi A, Tsuboi A, Kurata M, Fukuo K, Kazumi T. Carotid Intima-Media Thickness and Visit-to-Visit HbA1c Variability Predict Progression of Chronic Kidney Disease in Type 2 Diabetic Patients with Preserved Kidney Function. Journal of Diabetes Research. 2016;2016 (no pagination).

113. Teliti M, Cogni G, Sacchi L, Dagliati A, Marini S, Tibollo V, et al. Risk factors for the development of micro-vascular complications of type 2 diabetes in a single-centre cohort of patients. Diabetes & Vascular Disease Research. 2018;15(5):424-32.

114. Thyagarajan B, Howard AG, Durazo-Arvizu R, Eckfeldt JH, Gellman MD, Kim RS, et al. Analytical and biological variability in biomarker measurement in the Hispanic Community Health Study/Study of Latinos. Clinica Chimica Acta. 2016;463:129-37.

115. Tjaden AH, Edelstein S, Arslanian SA, Barengolts E, Caprio S, Cree-Green M, et al. Reproducibility of glycemic measures among youth and adults with impaired glucose tolerance (IGT) or recently diagnosed type 2 diabetes (T2D) in the restoring insulin secretion (RISE) study. Diabetes Conference: 79th Scientific Sessions of the American Diabetes Association, ADA. 2019;68(Supplement 1).

116. Trape J, Aliart ML, Brunet M, Dern E, Abadal E, Queralto JM. Reference change value for HbA1c in patients with type 2 diabetes mellitus. Clinical Chemistry & Laboratory Medicine. 2000;38(12):1283-7.

117. Tseng JY, Chen HH, Huang KC, Hsu SP, Chen CC. Effect of mean HbA1c on the association of HbA1c variability and all-cause mortality in patients with type 2 diabetes. Diabetes, Obesity and Metabolism. 2020;22(4):680-7.

118. Tsuboi A, Takenouchi A, Kurata M, Fukuo K, Kazumi T. Postmeal triglyceridemia and variability of HbA1c and postmeal glycemia were predictors of annual decline in estimated glomerular filtration rate in type 2 diabetic patients with different stages of nephropathy. Journal of Diabetes and Metabolic Disorders. 2017;16(1).

119. Tsuchiya T, Saisho Y, Murakami R, Watanabe Y, Inaishi J, Itoh H. Relationship between daily and visit-to-visit glycemic variability in patients with type 2 diabetes. Endocrine journal. 2020;08.

120. Tynjala A, Forsblom C, Harjutsalo V, Groop PH, Gordin D. HbA1c variability is associated with arterial stiffness in type 1 diabetes. Diabetologia. 2020;63(SUPPL 1):S441-S2.

121. Ucar F, Erden G, Ginis Z, Ozturk G, Sezer S, Gurler M, et al. Estimation of biological variation and reference change value of glycated hemoglobin (HbA1c) when two analytical methods are used. Clinical Chemistry. 2013;59(10):A87.

122. Virk SA, Donaghue KC, Cho YH, Benitez-Aguirre P, Hing S, Pryke A, et al. Association Between HbA1c Variability and Risk of Microvascular Complications in Adolescents With Type 1 Diabetes. The Journal of Clinical Endocrinology & Metabolism. 2016;101(9):3257-63.

123. Waden J, Forsblom C, Thorn LM, Gordin D, Saraheimo M, Groop P-H. A1C variability predicts incident cardiovascular events, microalbuminuria, and overt diabetic nephropathy in patients with type 1 diabetes. Diabetes. 2009;58(11):2649.

124. Walker GS, Cunningham SG, Sainsbury CAR, Jones GC. HbA1c variability is associated with increased mortality and earlier hospital admission in people with Type 1 diabetes. Diabetic Medicine. 2017;34(11):1541-5.

125. Watts GF, Morris RW, Goodland FC, Kubal C, Shaw KM. Serum fructosamine and glycosylated haemoglobin in the monitoring of glycaemic control in insulin-dependent diabetic outpatients. Practical Diabetes. 1989;6(4):159-63.

126. Whitsel EA, Nguyen QC, C MS, Tabor JW, Cuthbertson CC, Wener MH, et al. Dried capillary whole blood spot-based hemoglobin A1C, fasting glucose, and diabetes prevalence in a nationally representative population of young U.S. adults: Add health, wave IV. Circulation Conference: Epidemiology and Prevention/Physical Activity, Nutrition and Metabolism. 2012;125(10 SUPPL. 1).

127. Wightman SS, Sainsbury CAR, Jones GC. Visit-to-visit HbA1c variability and systolic blood pressure (SBP) variability are significantly and additively associated with mortality in individuals with type 1 diabetes: An observational study. Diabetes Obes Metab. 2018;20(4):1014-7.

128. Yang CY, Su PF, Hung JY, Ou HT, Kuo S. Comparative predictive ability of visit-to-visit HbA1c variability measures for microvascular disease risk in type 2 diabetes. Cardiovascular Diabetology. 2020;19(1).

129. Yang Y, Lee EY, Cho JH, Park YM, Ko SH, Yoon KH, et al. Cardiovascular autonomic neuropathy predicts higher hba1c variability in subjects with type 2 diabetes mellitus. Diabetes and Metabolism Journal. 2018;42(6):496-512.

130. Yang YF, Li TC, Li CI, Liu CS, Lin WY, Yang SY, et al. Visit-to-Visit Glucose Variability Predicts the Development of End-Stage Renal Disease in Type 2 Diabetes: 10-Year Follow-Up of Taiwan Diabetes Study. Medicine. 2015;94(44):e1804.

131. Yang HK, Kang B, Lee SH, Yoon KH, Hwang BH, Chang K, et al. Association between hemoglobin A1c variability and subclinical coronary atherosclerosis in subjects with type 2 diabetes. Journal of Diabetes & its Complications. 2015;29(6):776-82.

132. Zhong VW, Juhaeri J, Cole SR, Shay CM, Gordon-Larsen P, Kontopantelis E, et al. HbA1C variability and hypoglycemia hospitalization in adults with type 1 and type 2 diabetes: A nested case-control study. Journal of diabetes and its complications. 2018;32(2):203-9.
