# Supplementary material for: Within-subject variation of HbA1c: A systematic review and meta-analysis
Source: PLoS One. 2023 Aug 2;18(8):e0289085. doi: 10.1371/journal.pone.0289085 (PMC10395823; doi:10.1371/journal.pone.0289085)
Supplement: Peer review history

# Peer review: Decision Letter 0

**DOI:** 10.1371/journal.pone.0289085.r001

**Authors:** Ferdinando Carlo Sasso

18 May 2023

PONE-D-23-07141Within-subject variation of HbA1c: A systematic review and meta-analysisPLOS ONE

Dear Dr. Gough,

Thank you for submitting your manuscript to PLOS ONE. After careful consideration, we feel that it has merit but does not fully meet PLOS ONE’s publication criteria as it currently stands. Therefore, we invite you to submit a revised version of the manuscript that addresses the points raised during the review process.

Please submit your revised manuscript by Jul 02 2023 11:59PM. If you will need more time than this to complete your revisions, please reply to this message or contact the journal office at plosone@plos.org. When you're ready to submit your revision, log on to https://www.editorialmanager.com/pone/ and select the 'Submissions Needing Revision' folder to locate your manuscript file.

Please include the following items when submitting your revised manuscript:

- A rebuttal letter that responds to each point raised by the academic editor and reviewer(s). You should upload this letter as a separate file labeled 'Response to Reviewers'.
- A marked-up copy of your manuscript that highlights changes made to the original version. You should upload this as a separate file labeled 'Revised Manuscript with Track Changes'.
- An unmarked version of your revised paper without tracked changes. You should upload this as a separate file labeled 'Manuscript'.

If you would like to make changes to your financial disclosure, please include your updated statement in your cover letter. Guidelines for resubmitting your figure files are available below the reviewer comments at the end of this letter.

If applicable, we recommend that you deposit your laboratory protocols in protocols.io to enhance the reproducibility of your results. Protocols.io assigns your protocol its own identifier (DOI) so that it can be cited independently in the future. For instructions see: https://journals.plos.org/plosone/s/submission-guidelines#loc-laboratory-protocols. Additionally, PLOS ONE offers an option for publishing peer-reviewed Lab Protocol articles, which describe protocols hosted on protocols.io. Read more information on sharing protocols at https://plos.org/protocols?utm_medium=editorial-email&utm_source=authorletters&utm_campaign=protocols.

We look forward to receiving your revised manuscript.

Kind regards,

Ferdinando Carlo Sasso, PhD, MD

Academic Editor

PLOS ONE

Journal Requirements:

When submitting your revision, we need you to address these additional requirements.

1. Please ensure that your manuscript meets PLOS ONE's style requirements, including those for file naming. The PLOS ONE style templates can be found at 

https://journals.plos.org/plosone/s/file?id=wjVg/PLOSOne_formatting_sample_main_body.pdf and 

https://journals.plos.org/plosone/s/file?id=ba62/PLOSOne_formatting_sample_title_authors_affiliations.pdf

2. We note that you have indicated that data from this study are available upon request. PLOS only allows data to be available upon request if there are legal or ethical restrictions on sharing data publicly. For more information on unacceptable data access restrictions, please see http://journals.plos.org/plosone/s/data-availability#loc-unacceptable-data-access-restrictions. 

In your revised cover letter, please address the following prompts:

a) If there are ethical or legal restrictions on sharing a de-identified data set, please explain them in detail (e.g., data contain potentially sensitive information, data are owned by a third-party organization, etc.) and who has imposed them (e.g., an ethics committee). Please also provide contact information for a data access committee, ethics committee, or other institutional body to which data requests may be sent.

b) If there are no restrictions, please upload the minimal anonymized data set necessary to replicate your study findings as either Supporting Information files or to a stable, public repository and provide us with the relevant URLs, DOIs, or accession numbers. For a list of acceptable repositories, please see http://journals.plos.org/plosone/s/data-availability#loc-recommended-repositories.

We will update your Data Availability statement on your behalf to reflect the information you provide.

3. Please remove your figures from within your manuscript file, leaving only the individual TIFF/EPS image files, uploaded separately. These will be automatically included in the reviewers’ PDF.

4. Please include captions for your Supporting Information files at the end of your manuscript, and update any in-text citations to match accordingly. Please see our Supporting Information guidelines for more information: http://journals.plos.org/plosone/s/supporting-information. 

5. Please review your reference list to ensure that it is complete and correct. If you have cited papers that have been retracted, please include the rationale for doing so in the manuscript text, or remove these references and replace them with relevant current references. Any changes to the reference list should be mentioned in the rebuttal letter that accompanies your revised manuscript. If you need to cite a retracted article, indicate the article’s retracted status in the References list and also include a citation and full reference for the retraction notice.

Additional Editor Comments:

Please, address all issues raised by Reviewers.

[Note: HTML markup is below. Please do not edit.]

Reviewers' comments:

Reviewer's Responses to Questions

**Comments to the Author**

1. Is the manuscript technically sound, and do the data support the conclusions?

The manuscript must describe a technically sound piece of scientific research with data that supports the conclusions. Experiments must have been conducted rigorously, with appropriate controls, replication, and sample sizes. The conclusions must be drawn appropriately based on the data presented. 

Reviewer #1: Yes

Reviewer #2: Yes

**********

2. Has the statistical analysis been performed appropriately and rigorously? 

Reviewer #1: Yes

Reviewer #2: Yes

**********

3. Have the authors made all data underlying the findings in their manuscript fully available?

The PLOS Data policy requires authors to make all data underlying the findings described in their manuscript fully available without restriction, with rare exception (please refer to the Data Availability Statement in the manuscript PDF file). The data should be provided as part of the manuscript or its supporting information, or deposited to a public repository. For example, in addition to summary statistics, the data points behind means, medians and variance measures should be available. If there are restrictions on publicly sharing data—e.g. participant privacy or use of data from a third party—those must be specified.

Reviewer #1: Yes

Reviewer #2: Yes

**********

4. Is the manuscript presented in an intelligible fashion and written in standard English?

PLOS ONE does not copyedit accepted manuscripts, so the language in submitted articles must be clear, correct, and unambiguous. Any typographical or grammatical errors should be corrected at revision, so please note any specific errors here.

Reviewer #1: Yes

Reviewer #2: Yes

**********

5. Review Comments to the Author

Please use the space provided to explain your answers to the questions above. You may also include additional comments for the author, including concerns about dual publication, research ethics, or publication ethics. (Please upload your review as an attachment if it exceeds 20,000 characters)

Reviewer #1: I read with great interest “Within-subject variation of HbA1c: A systematic review and meta-analysis” by Alex Goughet al.

Paper design is fine. The article is logically divided into sections and subsections. Statistical analysis is well performed.

Comments:

1. The manuscript has been uploaded twice; I am reviewing only the first one.

2. Line 306-308: this may be due to different levels of insulin resistance, more than caloric intake. Please discuss.

3. The increased HbA1c variability is integrated in clinical practice with flash glucose monitoring. Moreover, in type 1 diabetes, the increasing role of continuous glucose monitoring associated to insulin pump have brought to a new concept of glucose variability which is beyond the role played by HbA1c. Please discuss it.

4. Glycaemic variability through HbA1c is burdened by a missing, which is the number of hypoglycaemic events that can occur and that can be masked by a period in hyperglycaemia. Please report, if available, data regarding hypoglycaemia and HbA1c variability.

Reviewer #2: Dear Editor, I’ve read with great interest the draft “Within-subject variation of HbA1c: A systematic review and meta-analysis”, by Alex Gough et al. However, some issues need to be raised.

- Line 125: please, define all the acronyms throughout the entire text.

- Line 335: Some important biochemical mechanisms may explain the association of HbA1c variability with all cause of mortality. I would suggest to briefly comment this point.

- Line 335: Moreover, a strict glycemic control may play a cardioprotective effect (doi: 10.1016/j.diabres.2021.108959.).

- A Conclusion section is needed to better describe the strengths of the draft.

**********

6. PLOS authors have the option to publish the peer review history of their article (what does this mean?). If published, this will include your full peer review and any attached files.

If you choose “no”, your identity will remain anonymous but your review may still be made public.

**Do you want your identity to be public for this peer review?** For information about this choice, including consent withdrawal, please see our Privacy Policy.

Reviewer #1: No

Reviewer #2: No

**********

[NOTE: If reviewer comments were submitted as an attachment file, they will be attached to this email and accessible via the submission site. Please log into your account, locate the manuscript record, and check for the action link "View Attachments". If this link does not appear, there are no attachment files.]

While revising your submission, please upload your figure files to the Preflight Analysis and Conversion Engine (PACE) digital diagnostic tool, https://pacev2.apexcovantage.com/. PACE helps ensure that figures meet PLOS requirements. To use PACE, you must first register as a user. Registration is free. Then, login and navigate to the UPLOAD tab, where you will find detailed instructions on how to use the tool. If you encounter any issues or have any questions when using PACE, please email PLOS at figures@plos.org. Please note that Supporting Information files do not need this step.

# Author comment: Author response to Decision Letter 0

**DOI:** 10.1371/journal.pone.0289085.r002

21 Jun 2023

Dear Dr Chenette, 

Thank you for the opportunity to revise my paper, “Within-subject variation of HbA1c: A systematic review and meta-analysis” (PONE-D-23-07141). 

All of the points raised by reviewers have been addressed:

1. “We note that you have indicated that data from this study are available upon request. PLOS only allows data to be available upon request if there are legal or ethical restrictions on sharing data publicly.”

The data extraction sheet has been uploaded. However, all data in the paper is derived from published sources, so all data is publicly available. 

2. “Please remove your figures from within your manuscript file, leaving only the individual TIFF/EPS image files, uploaded separately.”

This has been done.

3. “Please include captions for your Supporting Information files at the end of your manuscript, and update any in-text citations to match accordingly.” This has been done. 

4. “Please review your reference list to ensure that it is complete and correct. If you have cited papers that have been retracted, please include the rationale for doing so in the manuscript text, or remove these references and replace them with relevant current references.”

Some typographical errors have been corrected, and three references have been corrected in which the incorrect reference has been inserted when using Endnote software (62 (Jang 2019_, 122, (Virk 2016) and 127 (Wightman 2018)). One paper appears to be flagged as retracted, but this is actually a set of conference proceedings in which one abstract was withdrawn. The abstract cited in this study, (83, (Nagl 2014)) is unaffected. 

Reviewer 1

2. “Line 306-308: this may be due to different levels of insulin resistance, more than caloric intake. Please discuss.” 

Added “This may be due to different levels of insulin resistance, more than variation in caloric intake”

3.” The increased HbA1c variability is integrated in clinical practice with flash glucose monitoring. Moreover, in type 1 diabetes, the increasing role of continuous glucose monitoring associated to insulin pump have brought to a new concept of glucose variability which is beyond the role played by HbA1c. Please discuss it.”

4. “Glycaemic variability through HbA1c is burdened by a missing, which is the number of hypoglycaemic events that can occur and that can be masked by a period in hyperglycaemia. Please report, if available, data regarding hypoglycaemia and HbA1c variability.”

Regarding points 3 and 4. This paper is about variability in measured HbA1c not the adequacy of HbA1c as a measure of glucose control nor is the occurrence of hypoglycaemia the subject of the paper and so these comments are out of the scope of this paper. 

However, the following has been added:

“HbA1c may not be an ideal measure of glycaemic control because it does not capture hypoglycaemic events, in the future it may be replaced by flash glucose monitoring for some patients, but at present it is the main parameter used to assess glycaemic control and is likely to remain so for most patients.” 

Reviewer 2

“- Line 125: please, define all the acronyms throughout the entire text.”

The text has been reviewed and the following definitions added

within-individual coefficient of variation (CVi)

The European Federation of Clinical Chemistry and Laboratory Medicine (EFLM)

C-reactive protein (CRP)

National Institute for Healthcare and excellence (NICE)

Quality Assessment of Diagnostic Accuracy Studies (QUADAS tool).

“- Line 335: Some important biochemical mechanisms may explain the association of HbA1c variability with all cause of mortality. I would suggest to briefly comment this point.”

The following has been added: “due to a variety of mechanisms such as endothelial dysfunction, increased oxidative stress and increased release of cytokines”

“- Line 335: Moreover, a strict glycemic control may play a cardioprotective effect (doi: 10.1016/j.diabres.2021.108959.).”

The following has been added:“- A Conclusion section is needed to better describe the strengths of the draft.”

The following has been added:

“Conclusion

This comprehensive systematic review of estimates of HbA1c variability includes data from 111 papers. We provide separate estimates of HbA1c variability in healthy and diabetic populations. We also observe a positive correlation between mean HbA1c and within-individual variability.”

Thank you to the reviewers for their positive and helpful comments which help to strengthen this paper. I hope these revisions meet with your approval. 

Yours sincerely,

Alex Gough MA VetMB MRCVS

Institute of Applied Health Research

University of Birmingham

# Peer review: Decision Letter 1

**DOI:** 10.1371/journal.pone.0289085.r003

**Authors:** Ferdinando Carlo Sasso

11 Jul 2023

Within-subject variation of HbA1c: A systematic review and meta-analysis

PONE-D-23-07141R1

Dear Dr. Gough,

We’re pleased to inform you that your manuscript has been judged scientifically suitable for publication and will be formally accepted for publication once it meets all outstanding technical requirements.

Within one week, you’ll receive an e-mail detailing the required amendments. When these have been addressed, you’ll receive a formal acceptance letter and your manuscript will be scheduled for publication.

An invoice for payment will follow shortly after the formal acceptance. To ensure an efficient process, please log into Editorial Manager at http://www.editorialmanager.com/pone/, click the 'Update My Information' link at the top of the page, and double check that your user information is up-to-date. If you have any billing related questions, please contact our Author Billing department directly at authorbilling@plos.org.

If your institution or institutions have a press office, please notify them about your upcoming paper to help maximize its impact. If they’ll be preparing press materials, please inform our press team as soon as possible -- no later than 48 hours after receiving the formal acceptance. Your manuscript will remain under strict press embargo until 2 pm Eastern Time on the date of publication. For more information, please contact onepress@plos.org.

Kind regards,

Ferdinando Carlo Sasso, PhD, MD

Academic Editor

PLOS ONE

Additional Editor Comments (optional):

No further comments

Reviewers' comments:

Reviewer's Responses to Questions

**Comments to the Author**

1. If the authors have adequately addressed your comments raised in a previous round of review and you feel that this manuscript is now acceptable for publication, you may indicate that here to bypass the “Comments to the Author” section, enter your conflict of interest statement in the “Confidential to Editor” section, and submit your "Accept" recommendation.

Reviewer #1: All comments have been addressed

Reviewer #2: All comments have been addressed

**********

2. Is the manuscript technically sound, and do the data support the conclusions?

The manuscript must describe a technically sound piece of scientific research with data that supports the conclusions. Experiments must have been conducted rigorously, with appropriate controls, replication, and sample sizes. The conclusions must be drawn appropriately based on the data presented. 

Reviewer #1: Yes

Reviewer #2: (No Response)

**********

3. Has the statistical analysis been performed appropriately and rigorously? 

Reviewer #1: Yes

Reviewer #2: Yes

**********

4. Have the authors made all data underlying the findings in their manuscript fully available?

The PLOS Data policy requires authors to make all data underlying the findings described in their manuscript fully available without restriction, with rare exception (please refer to the Data Availability Statement in the manuscript PDF file). The data should be provided as part of the manuscript or its supporting information, or deposited to a public repository. For example, in addition to summary statistics, the data points behind means, medians and variance measures should be available. If there are restrictions on publicly sharing data—e.g. participant privacy or use of data from a third party—those must be specified.

Reviewer #1: Yes

Reviewer #2: Yes

**********

5. Is the manuscript presented in an intelligible fashion and written in standard English?

PLOS ONE does not copyedit accepted manuscripts, so the language in submitted articles must be clear, correct, and unambiguous. Any typographical or grammatical errors should be corrected at revision, so please note any specific errors here.

Reviewer #1: Yes

Reviewer #2: Yes

**********

6. Review Comments to the Author

Please use the space provided to explain your answers to the questions above. You may also include additional comments for the author, including concerns about dual publication, research ethics, or publication ethics. (Please upload your review as an attachment if it exceeds 20,000 characters)

Reviewer #1: The authors appropriately answered to all the issues I raised, the manuscript can be further processed for publication.

Reviewer #2: (No Response)

**********

7. PLOS authors have the option to publish the peer review history of their article (what does this mean?). If published, this will include your full peer review and any attached files.

If you choose “no”, your identity will remain anonymous but your review may still be made public.

**Do you want your identity to be public for this peer review?** For information about this choice, including consent withdrawal, please see our Privacy Policy.

Reviewer #1: No

Reviewer #2: No

**********

# Editor report: Acceptance letter

**DOI:** 10.1371/journal.pone.0289085.r004

**Authors:** Ferdinando Carlo Sasso

19 Jul 2023

PONE-D-23-07141R1 

Within-subject Variation of HbA1c: A systematic review and meta-analysis 

Dear Dr. Gough:

I'm pleased to inform you that your manuscript has been deemed suitable for publication in PLOS ONE. Congratulations! Your manuscript is now with our production department. 

If your institution or institutions have a press office, please let them know about your upcoming paper now to help maximize its impact. If they'll be preparing press materials, please inform our press team within the next 48 hours. Your manuscript will remain under strict press embargo until 2 pm Eastern Time on the date of publication. For more information please contact onepress@plos.org.

If we can help with anything else, please email us at plosone@plos.org. 

Thank you for submitting your work to PLOS ONE and supporting open access. 

Kind regards, 

PLOS ONE Editorial Office Staff

on behalf of

Professor Ferdinando Carlo Sasso 

Academic Editor

PLOS ONE
